# Structural and Functional Insight of Sphingosine 1-Phosphate-Mediated Pathogenic Metabolic Reprogramming in Sickle Cell Disease

**DOI:** 10.1038/s41598-017-13667-8

**Published:** 2017-11-10

**Authors:** Kaiqi Sun, Angelo D’Alessandro, Mostafa H. Ahmed, Yujin Zhang, Anren Song, Tzu-Ping Ko, Travis Nemkov, Julie A. Reisz, Hongyu Wu, Morayo Adebiyi, Zhangzhe Peng, Jing Gong, Hong Liu, Aji Huang, Yuan Edward Wen, Alexander Q. Wen, Vladimir Berka, Mikhail V. Bogdanov, Osheiza Abdulmalik, Leng Han, Ah-lim Tsai, Modupe Idowu, Harinder S. Juneja, Rodney E. Kellems, William Dowhan, Kirk C. Hansen, Martin K. Safo, Yang Xia

**Affiliations:** 1 Department of Biochemistry and Molecular Biology, The University of Texas Health Science Center at Houston, Houston, TX 77030 USA; 2 Graduate School of Biomedical Science, The University of Texas Health Science Center at Houston, Houston, TX 77030 USA; 3 Department of Biochemistry and Molecular Genetics, University of Colorado School of Medicine, Aurora, CO 80045 USA; 4 Department of Medicinal Chemistry, and The Institute for Structural Biology, Drug Discovery and Development, School of Pharmacy, Virginia Commonwealth University, Richmond, VA 23298 USA; 5 Department of Nephrology, Xiangya Hospital, Central South University, Changsha, Hunan 410008 China; 6 Department of Internal Medicine-Hematology, The University of Texas Health Science Center at Houston, Houston, TX 77030 USA; 7 Division of Hematology, The Children’s Hospital of Philadelphia, Philadelphia, PA 19104 USA

## Abstract

Elevated sphingosine 1-phosphate (S1P) is detrimental in Sickle Cell Disease (SCD), but the mechanistic basis remains obscure. Here, we report that increased erythrocyte S1P binds to deoxygenated sickle Hb (deoxyHbS), facilitates deoxyHbS anchoring to the membrane, induces release of membrane-bound glycolytic enzymes and in turn switches glucose flux towards glycolysis relative to the pentose phosphate pathway (PPP). Suppressed PPP causes compromised glutathione homeostasis and increased oxidative stress, while enhanced glycolysis induces production of 2,3-bisphosphoglycerate (2,3-BPG) and thus increases deoxyHbS polymerization, sickling, hemolysis and disease progression. Functional studies revealed that S1P and 2,3-BPG work synergistically to decrease both HbA and HbS oxygen binding affinity. The crystal structure at 1.9 Å resolution deciphered that S1P binds to the surface of 2,3-BPG-deoxyHbA and causes additional conformation changes to the T-state Hb. Phosphate moiety of the surface bound S1P engages in a highly positive region close to α1-heme while its aliphatic chain snakes along a shallow cavity making hydrophobic interactions in the “switch region”, as well as with α2-heme like a molecular “sticky tape” with the last 3–4 carbon atoms sticking out into bulk solvent. Altogether, our findings provide functional and structural bases underlying S1P-mediated pathogenic metabolic reprogramming in SCD and novel therapeutic avenues.

## Introduction

Sickle cell disease (SCD) is a prevalent life-threatening hemoglobinopathy characterized by a point mutation in the β-chain of hemoglobin. The aggregation of polymers of mutated sickle hemoglobin (HbS) under deoxygenated conditions causes sickling, a fundamental pathogenic process of the disease^1^. Although SCD was discovered more than a century ago and identified as the “first molecular disease” in 1949^2^, it is extremely disappointing that hydroxyurea is currently the only FDA-approved treatment. Notably, increased oxidative stress is also found in sickle erythrocytes and linked with hemolysis and disease progression^3^. Therefore, identifying specific factors and signaling pathways that contribute to sickling and oxidative stress is essential to advance our understanding of this pathogenic process and develop novel strategies for the treatment of SCD.

Recently, through accurately measuring functional phenotypes that are the net result of genomic, transcriptomic and proteomic changes, metabolomics profiling have become particularly useful to study mature erythrocytes, where gene expression profiling is not an option due to lack of a nucleus and *de novo* protein synthesis machinery. It has led to the discovery of substantial metabolic alterations in SCD erythrocytes of humans^4,5^ and mice^6^ and implicated multiple therapeutic possibilities. For example, metabolomics screening revealed that circulating adenosine and erythrocyte 2,3-bisphosphoglycerate (2,3-BPG), an erythroid specific glycolytic intermediate and potent allosteric modulator of Hb, and S1P, a bioactive signaling molecule highly enriched in erythrocytes, are elevated in patients and mice with SCD^6,7^. Mechanistic studies revealed that adenosine signaling through the adenosine A2B receptor (ADORA2B) underlies increased erythrocyte 2,3-BPG^6^ and S1P^8^ in patients and mice with SCD. Additional studies showed that pharmacologic inhibition or shRNA knockdown of sphingosine kinase 1 (Sphk1), the major enzyme in the spingolipid metabolism pathway to produce S1P from sphingosine in erythrocytes, significantly attenuated sickling and other deadly complications^7^. Moreover, a recent study reported that increased S1P induces oxygen (O_2_) delivery to counteract tissue hypoxia by inducing 2,3-BPG production in healthy individuals at high altitude and in normal mice exposed to hypoxia, which revealed the beneficial role of elevated erythrocyte S1P in normal individuals^9^. However, it is puzzling why elevated S1P is detrimental in SCD. To solve this puzzle, here we demonstrated the genetic, functional, metabolic and structural mechanisms underlying why the beneficial adaptation to high altitude in healthy individuals via induction of S1P in normal erythrocytes is detrimental in sickle erythrocytes. In contrast to normal erythrocytes, we revealed that genetic deletion of Sphk1 in SCD has potent anti-sickling and anti-hemolysis effects by correcting pathogenic metabolic reprogramming, channeling glucose to pentose phosphosphate pathway (PPP) relative to glycolysis, lowering 2,3-BPG production and boosting NADPH/glutathione-mediated detoxification. These findings open new promising scenarios in the development of innovative mechanism-based therapies for SCD.

## Results

### Genetic evidence for the pathogenic role of elevated Sphk1 in SCD mice

To precisely asses the detrimental role and mechanisms of elevated S1P in SCD, we generated a strain of mice with humanized sickle Hb and Sphk1 deficiency by crossing the SCD Berkeley mice^10^ with *Sphk1* ^−/−^ mice^11^ (Supplementary Fig. 1a). The *SCD/Sphk1* ^−/−^ offspring were viable and lived to adulthood. PCR analysis confirmed that the *Sphk1* gene was deleted, and high-performance liquid chromatography (HPLC) analysis of Hb species reveals the presence of only HbS in *SCD/Sphk1* ^−/−^ mice (Supplementary Fig. 1b,c). In *SCD/Sphk1* ^−/−^erythrocytes, Sphk1 activity is undetectable; erythrocyte and plasma S1P levels also decreased dramatically (Supplementary Fig. 1d,e). The remaining plasma S1P is presumably derived from the sphingosine kinase 2 (Sphk2) isoform expressed in a variety of cells^12^, but not in mature erythrocytes due to lack of a nucleus. Moreover, upstream sphingolipids such as ceramides and ceramide 1-phosphates increased significantly in *SCD/Sphk1* ^−/−^ mice (Supplementary Fig. 1g,h). Together, these data demonstrate that we have successfully deleted Sphk1 in the SCD Berkeley mice.

**Figure 1:**
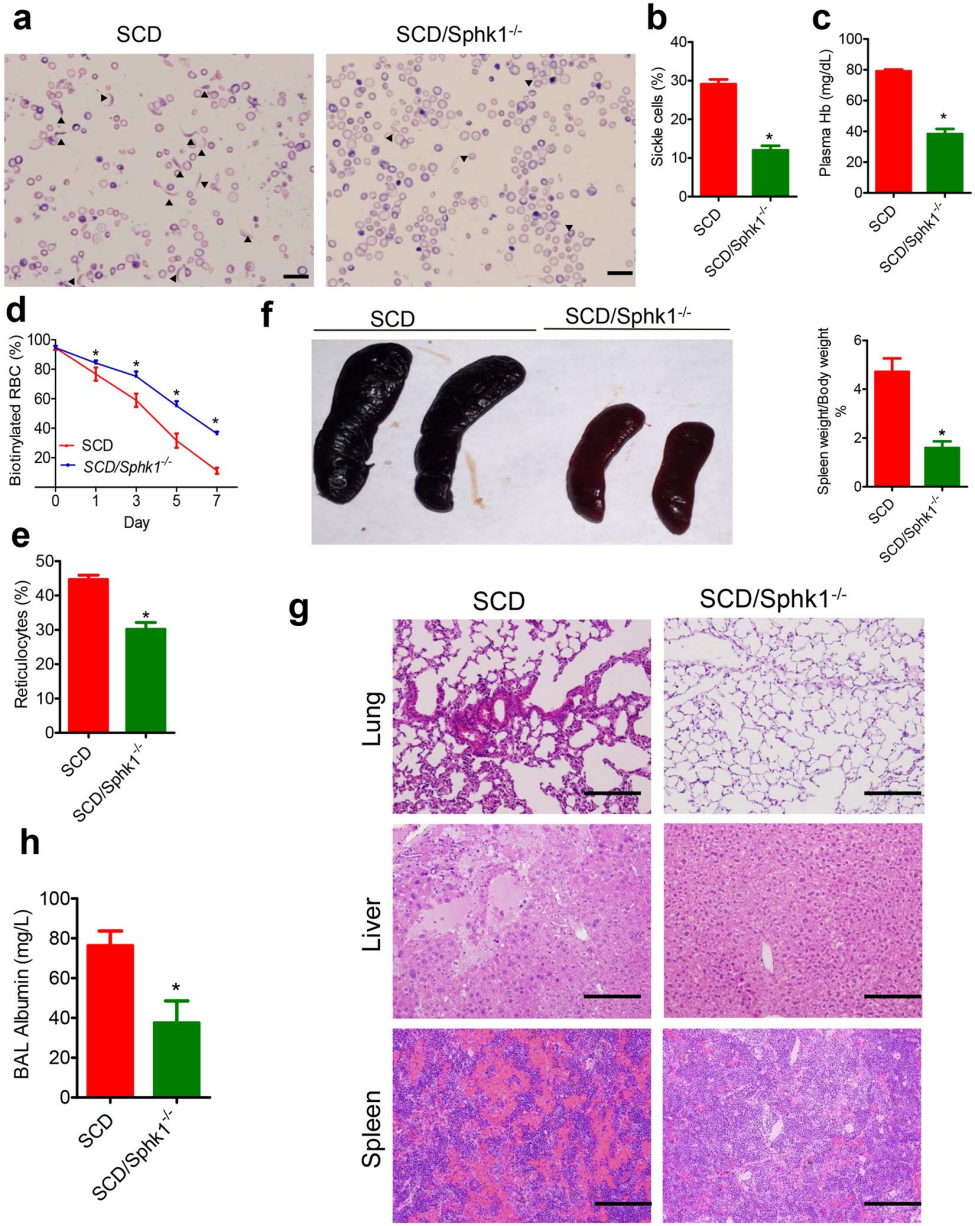
Genetic deletion of Sphk1 improves disease conditions in SCD Berkeley mice. (**a**) Representative pictures of blood smears from SCD and *SCD/Sphk1* ^−/−^ mice (magnification ×400). Percentage of sickle cells (**b**), plasma Hb (**c**) and reticulocytes (**e**) were significantly reduced while erythrocyte lifespan was significantly prolonged (**d**) by genetic deletion of Sphk1. Spleen size (**f**), H&E staining of spleens, livers, and lungs (**g**), and albumin concentrations in bronchial alveolar lavage (BAL) fluid (**h**) collected from SCD and *SCD/Sphk1* ^−/−^ mice. Values shown represent the mean ± SEM (n = 5-10); *p < 0.05 versus SCD, Student’s *t*-test. Scale bar: 20μm in blood smear pictures; 200μm in H&E staining pictures. Indicates sickled RBCs.

Next, we compared sickling in age and gender matched *SCD/Sphk1* ^−/−^ mice and SCD mice. Erythrocyte shape was much more uniform and organized in *SCD/Sphk1* ^−/−^ mice (Fig. 1a), and the percentage of irreversible sickle-shaped erythrocytes was significantly reduced (Fig. 1b). Because intravascular hemolysis is one of the major complications of SCD^1^, we assayed erythrocyte hemolysis by measuring plasma Hb concentration, which is significantly lower in *SCD/Sphk1* ^−/−^ mice (Fig. 1c). In agreement, we found the clear improvement of erythrocyte life-span in *SCD/Sphk1* ^−/−^ mice (Fig. 1d). Because of severe anemia, there is a large number of reticulocytes in SCD mice^10^, which were significantly reduced in *SCD/Sphk1* ^−/−^ mice (Fig. 1e). Complete blood count (CBC) analysis revealed higher total erythrocyte number, Hb concentration and hematocrit in *SCD/Sphk1* ^−/−^ mice (Supplementary Table 1). Moreover, the erythrocyte distribution width was also significantly reduced (Supplementary Table 1). Because S1P is a potent immune regulator^13^, we found that the peripheral white blood cell count was dramatically decreased in *SCD/Sphk1* ^−/−^ mice with both neutrophil and lymphocyte counts back to the normal range (Supplementary Table 1). Splenomegaly and multiple organ damage are the hallmarks of SCD progression^1^. Consistent with the above improvements, splenomegaly (Fig. 1f), congestion and damage in spleen, lungs and liver were also significantly improved in *SCD/Sphk1* ^−/−^ mice, as indicated by histology analysis (Fig. 1g; Supplementary Fig. 2). Albumin level in the bronchoalveolar lavage fluid was also significantly reduced (Fig. 1h), indicating less vascular leakage in the lungs of *SCD/Sphk1* ^−/−^ mice. Taken together, these data provide solid genetic evidence demonstrating that deletion of Sphk1 is beneficial in SCD.

### Enhanced erythrocyte pentose phosphate pathway and anti-oxidation capacity in *SCD/Sphk1*^−/−^ mice

**Figure 2:**
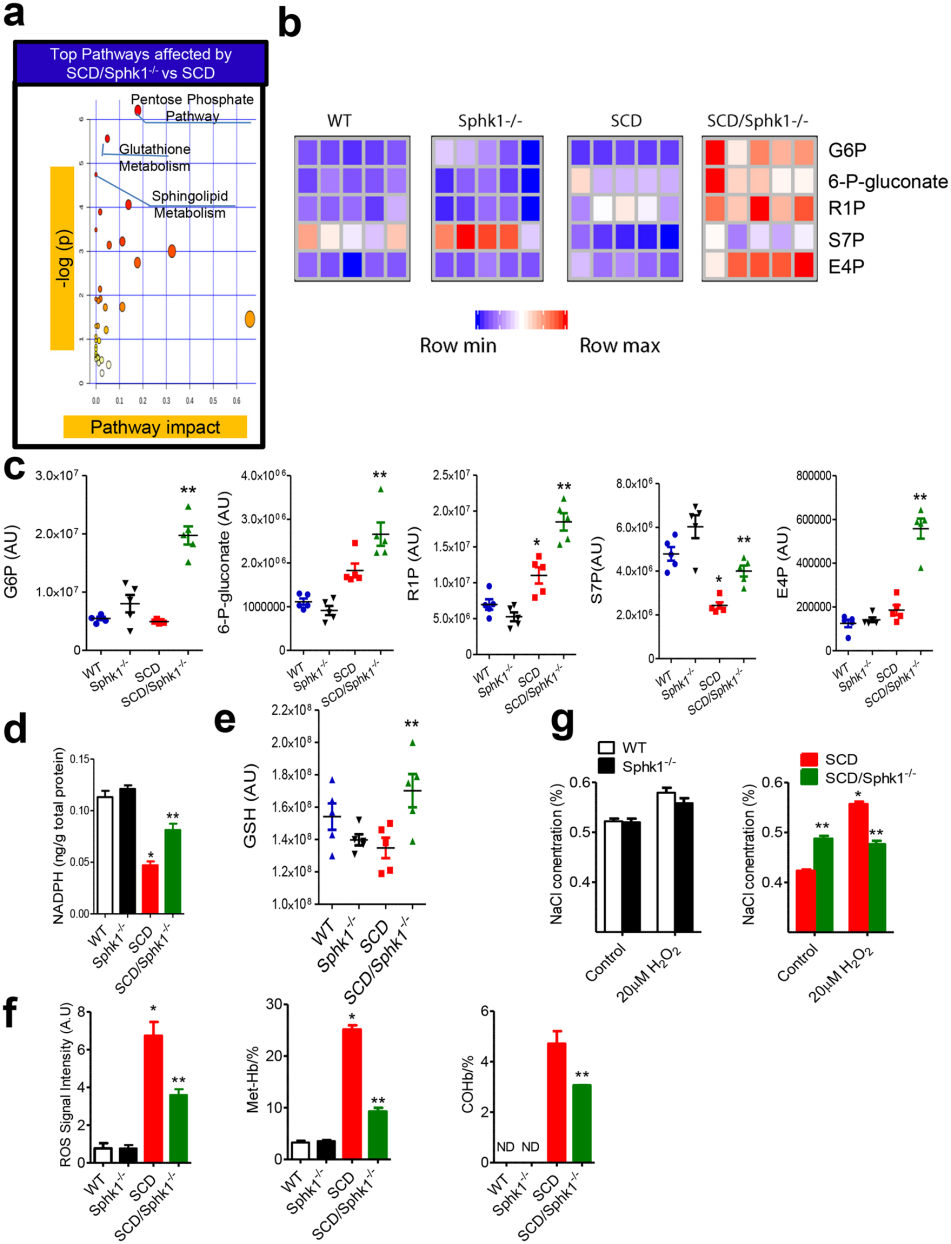
Enhanced pentose phosphate pathway and anti-oxidant capacity in *SCD/Sphk1* ^−/−^ mouse erythrocytes. (**a**) Top metabolic pathways affected by genetic deletion of Sphk1 in SCD mice. (**b**) Relative abundance of selected PPP metabolites in erythrocytes from WT, *Sphk1* ^−/−^, SCD and *SCD/Sphk1* ^−/−^ mice. (**c**) Intensity peak of selected PPP metabolites in erythrocytes from WT, *Sphk1* ^−/−^, SCD and *SCD/Sphk1* ^−/−^ mice detected by metabolomics screening. Levels of NADPH (**d**), GSH (**e**) and in erythrocytes from WT, *Sphk1* ^−/−^, SCD and *SCD/Sphk1* ^−/−^ mice. (**f**) ROS, MetHb and COHb levels in erythrocytes from WT, *Sphk1* ^−/−^, SCD and *SCD/Sphk1* ^−/−^ mice. (**g**) Resistance of WT, *Sphk1* ^−/−^, SCD and *SCD/Sphk1* ^−/−^ erythrocytes to osmolality-induced hemolysis with or without oxidative stress challenge. Values shown represent the mean ± SEM (n = 5); *p < 0.05 versus WT; **p < 0.05 versus SCD; Student’s *t*-test. G6P: Glucose 6-phosphate; 6-P-Gluconate: Gluconate-6-phosphate; R1P: Ribose 1-phosphate; E4P: Erythrose 4-phosphate; S7P: Sedoheptulose 7-phosphate; GSH: reduced glutathione.

Giving the solid genetic evidence of elevated Sphk1 contributing to sickling and disease progression^7^, we sought to further determine the molecular basis. Because erythrocytes lack nuclei and organelles, metabolic adaptation has a key role in erythrocyte homeostasis^14^. Therefore, we chose to exploit an unbiased high throughput metabolomic profiling to compare global metabolic changes in the erythrocytes among WT, *Sphk1* ^−/−^, SCD and *SCD/Sphk1* ^−/−^ mice. A total of 222 named metabolites were detected in over 9,000 features screened (Supplementary Data 1). Next, we performed an unbiased pathway-enrichment analysis using MetaboAnalyst^15^. First, genetic deletion of Sphk1 in normal mice did not affect erythrocyte metabolism other than the significantly decreased S1P levels in erythrocytes and plasma and increased ceramides (Supplementary Fig. 1e–g). However, a large portion of the 25 pathways identified were affected by Sphk1 deficiency in SCD Berkeley mice, and the top three metabolic pathways affected by genetic deletion of Sphk1 in SCD mice were pentose phosphate pathway (PPP), glutathione metabolism, and sphingolipid metabolism (Fig. 2a). Sphingolipid metabolism alteration validates the impact of Sphk1 deletion (Supplementary Fig. 1d–h). Moreover, we found substantially increased steady state levels of multiple intermediates of PPP including glucose 6-phosphate (G6P), gluconate-6-phosphate (6-P-gluconate), ribose 1-phosphate (R1P), erythrose 4-phosphate (E4P) and sedoheptulose 7-phosphate (S7P) in the erythrocytes of *SCD/Sphk1* ^−/−^ mice relative to SCD mice (Fig. 2b,c), suggesting that the PPP is significantly enhanced in *SCD/Sphk1* ^−/−^ erythrocytes. In agreement with enhanced PPP, we found increased NADPH, an important byproduct of this pathway, in *SCD/Sphk1* ^−/−^ erythrocytes (Fig. 2d). As such, reduced glutathione (GSH), a key NADPH-dependent antioxidant, was substantially elevated (Fig. 2e). Altogether, these data strongly suggest a decrease in oxidative stress in *SCD/Sphk1* ^−/−^ erythrocytes. Not surprisingly, we detected significantly lower ROS levels, MetHb and COHb in *SCD/Sphk1* ^−/−^ erythrocytes (Fig. 2f). Numerous studies have indicated that excessive oxidative stress in SCD leads to hemolysis and erythrocyte destruction^3^. Thus, we determined if deletion of Sphk1 increases resistance of SCD erythrocytes to hemolytic challenges induced by oxidative stress. After exposure to hydrogen peroxide (H_2_O_2_), *SCD/Sphk1* ^−/−^ erythrocytes had a significantly lower osmotic fragility with increased half-maximal effective concentrations (EC50) (Fig. 2g), consistent with increased GSH and NADPH-dependent antioxidant capacity.

### Reduced glycolysis and Hb-O_2_ binding affinity in *SCD/Sphk1*^−/−^ erythrocytes

**Figure 3:**
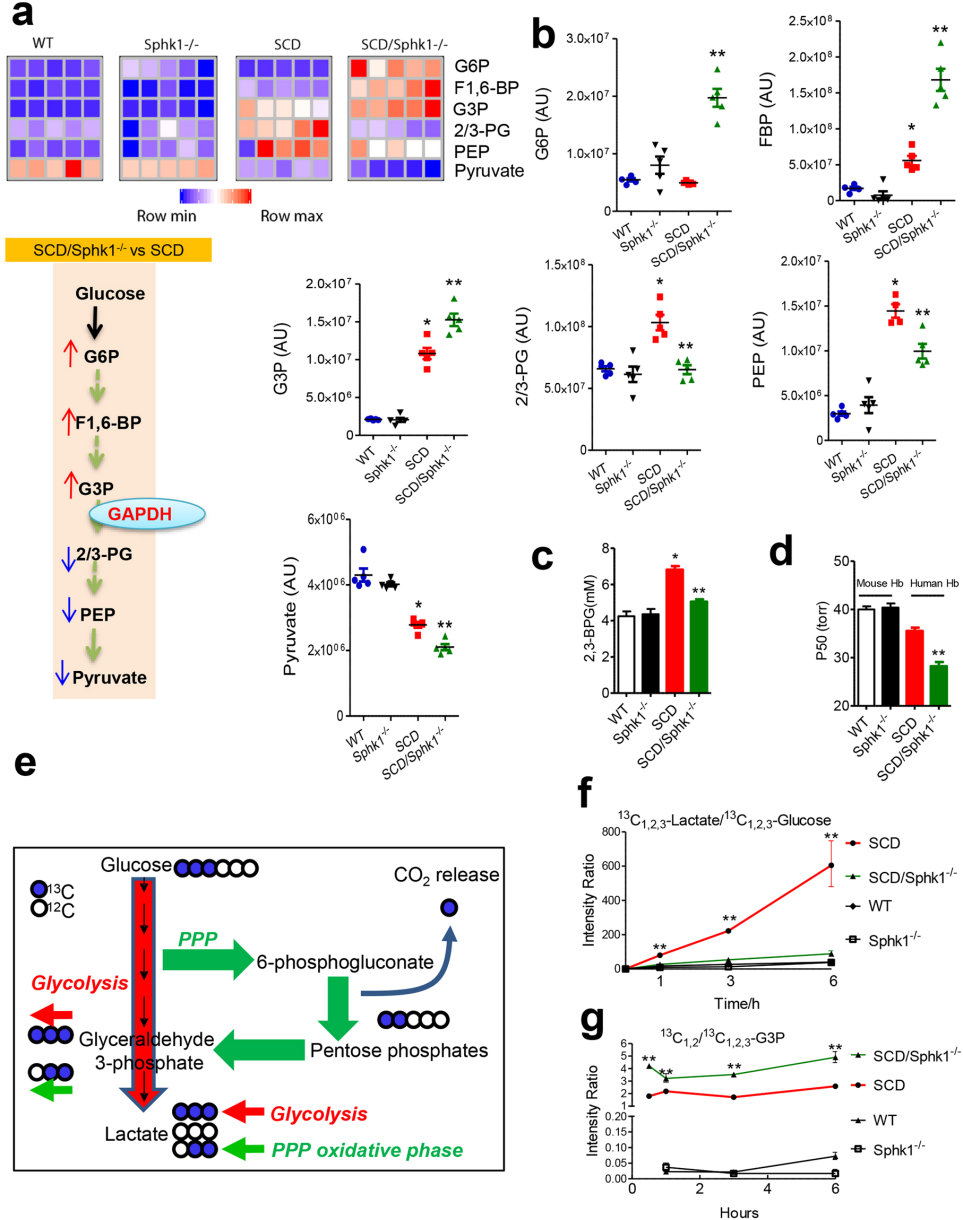
Genetic deletion of Sphk1 reduces glycolysis and O_2_ release and channels glucose flux to PPP in SCD erythrocytes. (**a**) Relative abundance of selected glycolysis metabolites in erythrocytes from WT, *Sphk1* ^−/−^, SCD and *SCD/Sphk1* ^−/−^ mice (upper); glycolysis is blocked at the step where G3P is metabolized by GAPDH in erythrocytes of *SCD/Sphk1* ^−/−^ mice compared to SCD mice (lower). (**b**) Intensity peak of selected glycolysis metabolites in erythrocytes from WT, *Sphk1* ^−/−^, SCD and *SCD/Sphk1* ^−/−^ mice detected by metabolomics screening. 2,3-BPG level (**c**) and P50 (**d**) in WT, *Sphk1* ^−/−^, SCD and *SCD/Sphk1* ^−/−^ mouse erythrocytes. (**e**) Schematic illustration of glucose metabolism flux detection using^13^C_1,2,3_-Glucose. Ratios of ^13^C_1,2,3_-Lactate/^13^C_1,2,3_-Glucose (**f**) and^13^C_2,3_-/^13^C_1,2,3_-G3P (**g**) in WT, *Sphk1* ^−/−^, SCD and *SCD/Sphk1* ^−/−^ mouse erythrocytes. Values shown represent the mean ± SEM (n = 5); *p < 0.05 versus WT; **p < 0.05 versus SCD; Student’s *t*-test. PPP: pentose phosphate pathway; G6P: Glucose 6-phosphate; FBP: Fructose 1,6-bisphosphate; G3P: Glyceraldehyde 3-phosphate; 2/3-PG: 2/3-Phosphoglyceric acid; PEP: Phosphoenolpyruvate; CO_2_: carbon dioxide.

Glucose in erythrocytes is metabolized through either PPP, to generate reducing equivalents to preserve redox homeostasis, or glycolysis, to produce ATP as an energy source^16^. Additionally, approximately 19~25% of the glucose is utilized to produce 2,3-BPG, a key allosteric regulator of Hb-O_2_ affinity, which derives from the Rapoport-Luebering branch of glycolysis^17^. Under high O_2_ saturation conditions, oxidative stress promotes PPP to generate NADPH. To deliver O_2_ efficiently while neutralizing excessive oxidative stress caused by a heavy load of O_2_, erythrocytes rely on a finely-tuned O_2_–dependent modulation of glucose metabolism^18–20^. Based on the enhanced PPP and glutathione metabolism in the erythrocytes of *SCD/Sphk1* ^−/−^ mice (Fig. 2), we sought to test if increased steady state levels of PPP intermediates in *SCD/Sphk1* ^−/−^ erythrocytes correspond to a decline of metabolic flux through glycolysis. First, we found significantly increased glycolytic intermediates including G6P, fructose 1,6-bisphosphate (FBP), glyceraldehyde 3-phosphate (G3P), 2/3-phosphoglyceric acid (2/3-PG), phosphoenolpyruvate (PEP) and pyruvate in SCD mouse erythrocytes compared to WT (Fig. 3a,b), confirming that glycolysis rather than the PPP is preferentially active in SCD mouse erythrocytes (Fig. 3a,b), which explains the compromised capacity to produce reducing equivalents (Fig. 2d) and preserve glutathione homeostasis in SCD (Fig. 2e). In addition, levels of these metabolites were not different between *Sphk1* ^−/−^ and WT erythrocytes. To our surprise, the levels of three upstream metabolites of glycolysis including G6P, FBP and G3P were increased in *SCD/Sphk1* ^−/−^ erythrocytes compared to SCD (Fig. 3a,b), suggesting a metabolic bottleneck downstream to G3P dehydrogenase (GAPDH)^21^. In contrast, the levels of three glycolytic intermediates downstream of G3P including 2/3-PG, PEP and pyruvate were significantly reduced in *SCD/Sphk1* ^−/−^ erythrocytes (Fig. 3a,b). More importantly, the levels of 2,3-BPG, an erythrocyte-specific metabolite contributing to sickling^6,22,23^ and an intermediate downstream of G3P, was increased in the SCD erythrocytes but decreased in those of *SCD/Sphk1* ^−/−^ mice (Fig. 3c).

Given the fact that 2,3-BPG decreases HbS-O_2_ binding affinity^22,24^ and in view of our findings that elevated Sphk1 contributes to increase 2,3-BPG production in sickle erythrocytes, we hypothesize that elevated Sphk1 underlies sickling by inducing 2,3-BPG, decreasing HbS-O2 binding affinity and thus increasing deoxy-HbS polymerization. To test this hypothesis, we measured the O_2_ equilibrium curve (OEC) by calculating the partial pressure of O_2_ required to produce 50% Hb-O_2_ saturation (P50), and found increased Hb-O_2_ binding affinity and thus reduced P50 in *SCD/Sphk1* ^−/−^ mouse erythrocytes (Fig. 3d). However, there was no difference between WT and *Sphk1* ^−/−^ erythrocytes. These findings indicate that decreased 2,3-BPG due to deficiency of Sphk1 results in increased Hb-O_2_ binding affinity and decreased deoxyHbS level, which support the observation of less sickling in *SCD/Sphk1* ^−/−^ mice (Fig. 1a). Altogether, the beneficial role of Sphk1 deficiency in anti-sickling and anti-hemolysis is strongly supported by metabolic rewiring in *SCD/Sphk1* ^−/−^ erythrocytes.

### Genetic deletion of Sphk1 channels glucose fluxes to PPP in SCD erythrocytes

Next, to provide direct mechanistic insight about intracellular glucose flux, we first assayed glucose uptake in WT, *Sphk1* ^−/−^, SCD and *SCD/Sphk1* ^−/−^ erythrocytes. Interestingly, although glucose uptake is significantly increased in the SCD Berkeley mouse erythrocytes compared to normal, which agrees with previous studies done in human SCD erythrocytes^5^, there was no difference between SCD and *SCD/Sphk1* ^−/−^ or WT and *Sphk1* ^−/−^ erythrocytes (Supplementary Fig. 3a), indicating that differences in PPP and glycolysis pathways in SCD and *SCD/Sphk1* ^−/−^ erythrocytes are not caused by variation in glucose uptake. In addition, we used the stable ^13^C_1,2,3-_glucose isotope to trace intracellular glucose metabolism through glycolysis and PPP in SCD and *SCD/Sphk1* ^−/−^ erythrocytes at different time points. Specifically, we investigated whether glycolysis or the PPP is the major contributor to the accumulation of G3P in *SCD/Sphk1* ^−/−^ mouse erythrocytes by determining the ratios of the isotopologues ^13^C_2,3_/^13^C_1,2,3_ of G3P (Fig. 3e). First, ^13^C_1,2,3_-lactate/^13^C_1,2,3_-glucose ratios were significantly increased in SCD but not in *SCD/Sphk1* ^−/−^ mouse erythrocytes in a time-dependent manner, indicating significantly increased metabolic flux through PPP in SCD mouse erythrocytes (Fig. 3f). In agreement, ratios of ^13^C_1,2,3_-6-P-gluconate, a PPP metabolite, to ^13^C_1,2,3_-glucose were significantly higher in *SCD/Sphk1* ^−/−^ mouse erythrocytes (Supplementary Fig. 3b). As expected, ratios of ^13^C_2,3_-G3P/^13^C_1,2,3_-G3P isotopologues were significantly higher in erythrocytes from *SCD/Sphk1* ^−/−^ erythrocytes (Fig. 3g), indicating that glucose flux through the PPP was enhanced.

### Sphk1 regulates GAPDH localization in SCD erythrocyte

**Figure 4:**
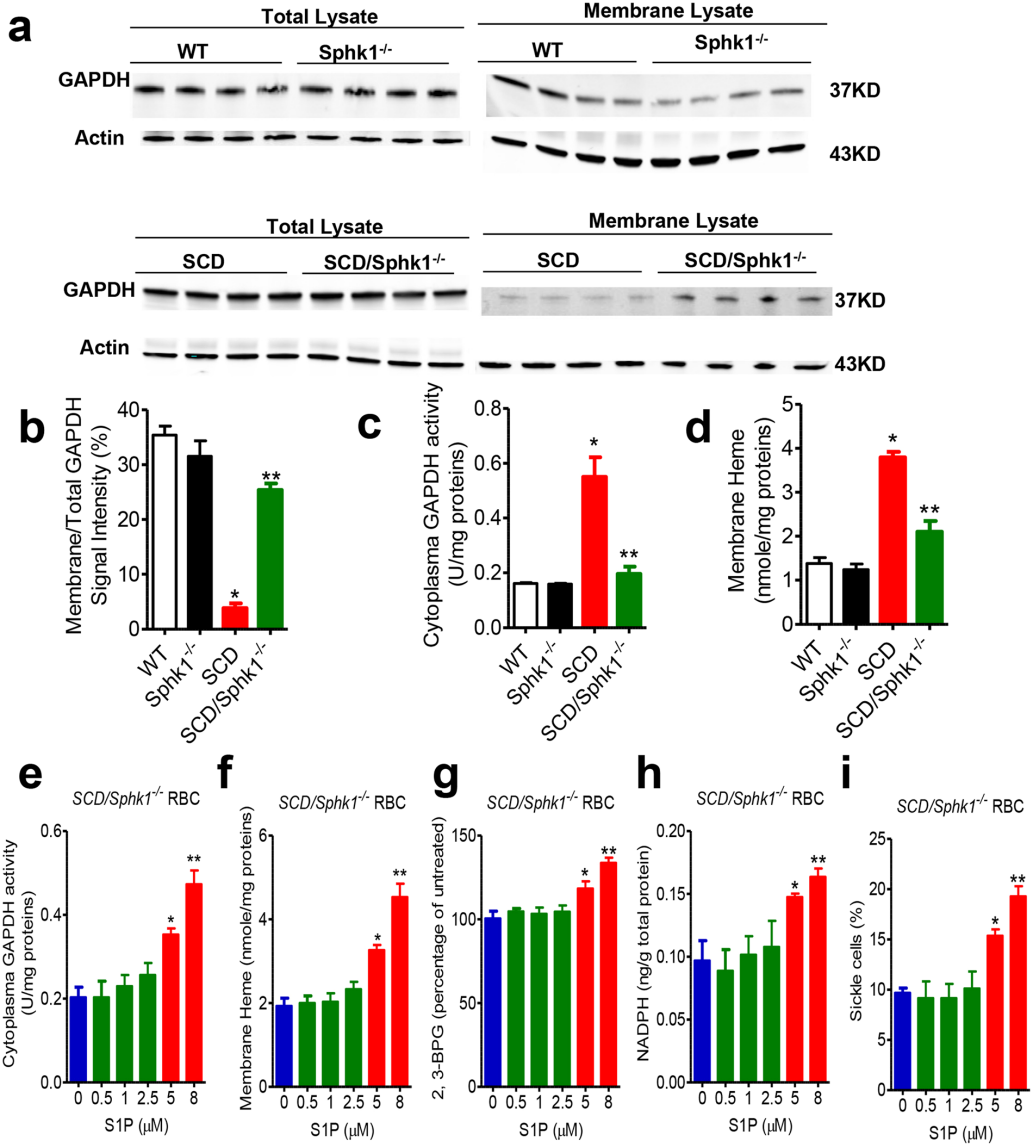
Sphk1-mediated production of S1P functions intracellularly to regulate GAPDH and Hb localization and subsequent metabolic consequences. (**a**,**b**) Total and membrane bound GAPDH protein levels in WT, *Sphk1* ^−/−^, SCD and *SCD/Sphk1* ^−/−^ mouse erythrocytes detected by western blot (cropped blots displayed, whole blots see supplementary information). Cytosolic GAPDH activity (**c**) and membrane bound heme (**d**) in WT, *Sphk1* ^−/−^, SCD and *SCD/Sphk1* ^−/−^ mouse erythrocytes. Cytosolic GAPDH activity (**e**), membrane bound heme (**f**), 2,3-BPG levels (**g**), NADPH levels (**h**) and percentage of sickled erythrocytes in *SCD/Sphk1* ^−/−^ mouse erythrocytes treated with different concentration of S1P. Values shown represent the mean ± SEM (n = 5); *p < 0.05 versus WT or 2.5 µM; **p < 0.05 versus SCD or 5 µM, Student’s *t*-test.

Under normoxia, erythrocyte glucose flux through glycolysis is limited by the inhibitory sequestration of glycolytic enzymes, including GAPDH, to the cytosolic domain of the membrane protein Band3 (cdB3)^20^. However, under hypoxia, deoxygenated Hb (deoxyHb) competes with glycolytic enzymes for binding to cdB3, which results in the release of those enzymes, thereby promoting glycolysis^18–20^. Recent studies have revealed that deoxyHbS disturbs normal coupling among erythrocyte O_2_ content, glycolysis and antioxidant capacity by increasing release of membrane anchored GAPDH to the cytosol^5^. Therefore, we investigated if elevated erythrocyte Sphk1-mediated increased S1P is involved in regulating the intracellular location of GAPDH by facilitating the binding of deoxyHbS to cdB3 and the release of GAPDH to the cytosol. Western blot results indicated no obvious difference in total amount of GAPDH (Fig. 4a,b). However, a significantly larger percentage of GAPDH in *SCD/Sphk1* ^−/−^ erythrocyte was found on the membrane (Fig. 4a,b). In agreement, cytosolic GAPDH activity was significantly reduced in *SCD/Sphk1* ^−/−^ erythrocytes compared to *SCD* (Fig. 4c). However, no significant difference of GAPDH localization and activity was found between WT and *Sphk1* ^−/−^ erythrocytes (Fig. 4a–c). Thus, genetic and biochemical evidence demonstrate that Sphk1 enhances release of membrane anchored GAPDH and increases cytosolic GAPDH activity in SCD erythrocytes. Next, to dissect the influence of non-specifically bound HbS, we isolated erythrocyte membrane ghosts from *SCD* and *SCD/Sphk1* ^−/−^ mice and inverted the membrane ghost on silicon beads and washed for 8 times with low-salt buffer. Then, we detected significantly higher levels of heme anchored on the ghost membrane of erythrocytes from SCD (Fig. 4d). Thus, these findings indicate that elevated Sphk1 is associated with enhanced HbS anchoring to membrane, and are consistent with the release of membrane bound GAPDH and increased cytosolic GAPDH activity.

S1P is the ligand to five G-protein coupled receptors. To test if extracellular or intracellular S1P is playing the major role in regulating Hb and GAPDH localization and erythrocyte metabolism, we treated *SCD/Sphk1* ^−/−^ erythrocytes with different concentrations of S1P to mimic conditions in SCD mice. Interestingly, S1P treatment up to 2.5 µM, which are sufficient to activate all of the S1P receptors, did not affect cytosolic GAPDH, membrane heme, 2,3-BPG and NADPH in *SCD/Sphk1* ^−/−^ mouse erythrocytes (Fig. 4e–h). However, when treated with higher concentrations of S1P that can lead to increase of intracellular S1P levels^25^, all of the above parameters showed dose-dependent increase (Fig. 4e–h). Moreover, we found that sickling of *SCD/Sphk1* ^−/−^ erythrocyte was induced not in low concentrations of S1P at but only in 5 and 8 µM (Fig. 4i). Thus, these data implicate that decreased sickling mediated by Sphk1 deficiency is independent of S1P receptors. Overall, we provide direct evidence that S1P functions intracellularly as a modulator promoting deoxy-HbS anchoring to the membrane and subsequently enhancing the release of membrane bound GADPH to the cytosol, which in turn leads to increased cytosolic GAPDH activity in SCD erythrocytes.

### Co-binding of 2,3-BPG and S1P to Hb is required for S1P-induced decrease in Hb-O_2_ affinity

**Figure 5:**
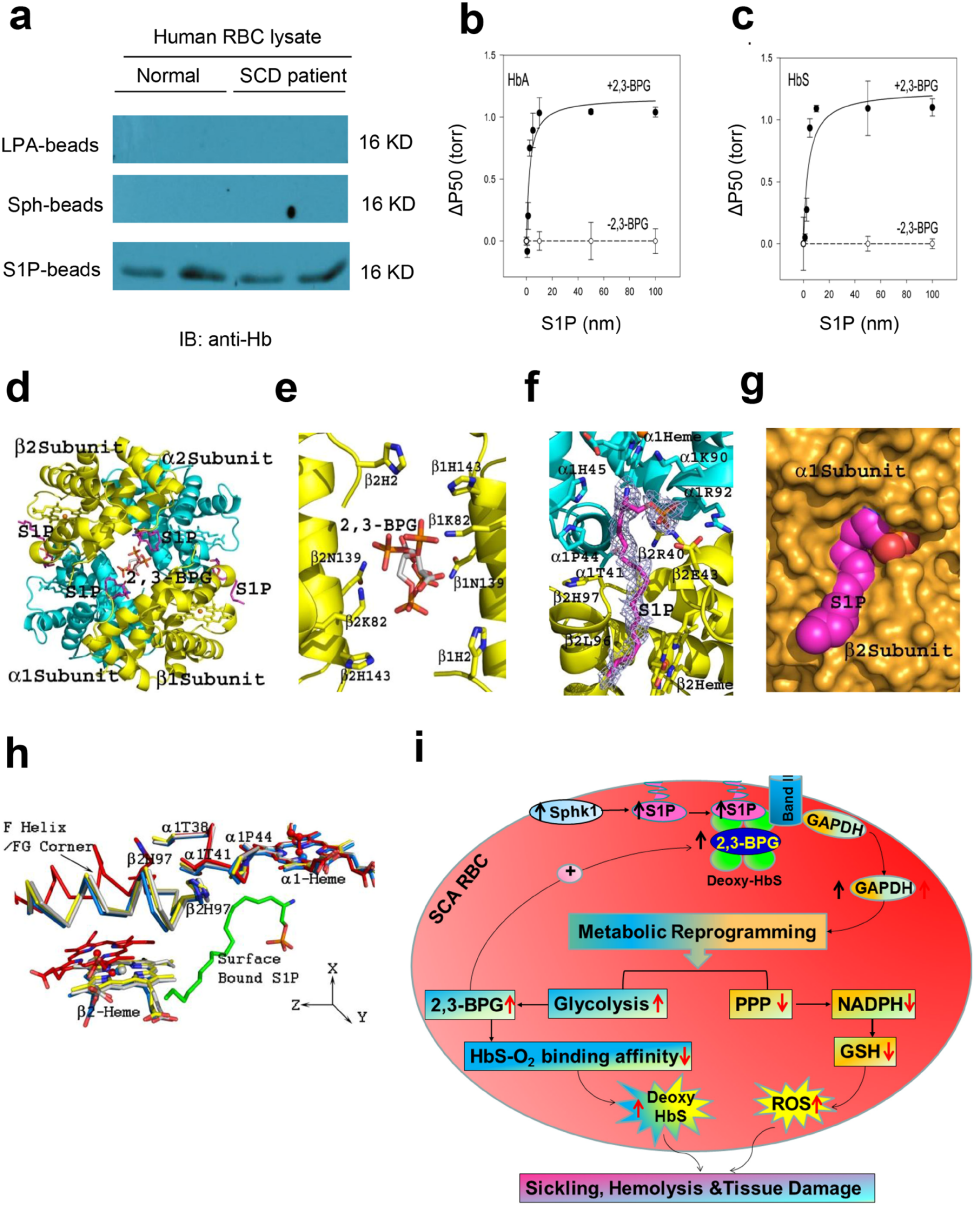
Functional and structural evidence for S1P binding to Hb and stabilization of deoxyHb in T-state. (**a**) Pull-down of Hb by lysophosphatidic acid (LPA), Sphingosine and S1P beads from normal human and SCD patient RBC lysates (cropped blots displayed, for full blots see supplementary information). S1P, at physiological and pathological molar ratios, induces further O_2_ release from HbA (**b**) and HbS (**c**) in the presence of 2,3-BPG. (**d**) Crystal structure of deoxyHbA in complex with 2,3-BPG (bound at the β-cleft), and S1P (bound both at the central water cavity and the protein surface). (**e**) Close view of 2,3-BPG binding at the β-cleft. (**f**–**h**) S1P binds to the surface of HbA in the presence of 2,3-BPG and induces further conformational change stabilizing the complex in T-state. (**i**) Working model: elevated erythrocyte Sphk1 activity increases production of S1P, which binds to deoxyHbS and facilitates deoxyHbS anchoring to membrane and release of GAPDH. Increased cytosolic GAPDH accelerates glycolysis and 2,3-BPG production while decreasing PPP and antioxidant production. Increased 2,3-BPG leads to more deoxyHbS and more sickling while decreased antioxidant causes more oxidative stress (ROS) and more hemolysis. Altogether, erythrocyte S1P induced by elevated Sphk1 activity leads to impaired metabolic reprogramming and thus underlies sickling, hemolysis and disease progression in SCD.

Since S1P directly induces HbS anchoring to membrane, we speculated that S1P binds to HbS and HbA and reduces Hb-O_2_ affinity as other phosphates do. Indeed, S1P-conjugated beads successfully pulled down HbA and HbS from normal and SCD patients erythrocyte lysates, while sphingosine or lysophosphatidic acid beads cannot (Fig. 5a), indicating that S1P directly and specifically binds to both human HbA and HbS in erythrocyte lysates. Next, to determine if S1P regulates Hb-O_2_ binding affinity, we assayed HbA and HbS O_2_ binding equilibrium curves in the absence or presence of different concentrations of S1P. To mimic the molar ratio of S1P to Hb from 1:2500 to 1:500 as seen in normal and sickle human erythrocytes, we used HbA or HbS at 10 µM with the concentrations of S1P ranging from 0 to 10 nM. Unexpectedly, S1P alone has no effect on Hb-O_2_ binding affinity. Realizing that there is very abundant 2,3-BPG is in erythrocytes which binds to deoxyHb, we tested if 2,3-BPG is required for S1P-mediated reduction in Hb-O_2_ affinity. P50 of purified HbA or HbS in the presence of 2,3-BPG along with S1P revealed that S1P decreased HbA and HbS-O_2_ binding affinity in a dose-dependent manner (Fig. 5b and c). Thus, we provide biochemical and functional evidence that S1P binds directly to Hb but requires co-binding of 2,3-BPG to decrease O_2_ binding affinity, presumably by further stabilizing deoxyHb and increasing its T-state character.

### X-ray crystallography reveals atomic level insight into S1P-Hb binding

Given the fact that structures (both tertiary and quaternary) of liganded or unliganded normal HbA and sickle HbS are identical even at the pathogenic βVal6 mutation site^26,27^ and that it is easier to crystalize HbA, we chose to determine the crystal structures of deoxyHbA in complex with S1P alone (deoxyHbA-S1P) or in combination with 2,3-BPG (deoxyHbA-S1P-BPG) (subsequently solved at 2.4 Å and 1.8 Å) to gain structural insight into the above described S1P-mediated functional/biological effects. The structures were determined by molecular replacement using the high resolution native deoxyHbA structure (PDB code: 2DN2)^28^. Expectedly, and consistent with published studies, we observed 2,3-BPG bound in two alternate conformations at the dyad axis of the β-cleft in the ternary deoxyHbA-S1P-2,3-BPG complex to tie together the two β-subunits via interactions with the residues βHis2, βLys82, βAsn139, and βHis143 from both β-subunits (intermolecular interactions) in symmetry-related fashion (Fig. 5d and e). In both the binary deoxyHbA-S1P and ternary deoxyHbA-S1P-2,3-BPG complexes, S1P was observed bound in the central water cavity, with the phosphate and the amide moieties located in a pocket formed by α1Lys99, α1His103, β1Asn108, β1Tyr35 and β1Gln131, while the flexible aliphatic long chain snaked toward the β-cleft making hydrophobic interactions with α1 Phe36, α1Ser35, β1Lys132, β1Gln131, β1Ala135, β1Val1 and β1His2 (Supplementary Fig. 4). However, while the ternary complex showed S1P bound in a symmetry-related fashion (Fig. 5d; Supplementary Fig. 4), the binding of S1P in the binary deoxyHbA-S1P complex was very weak (Supplementary Fig. 5), and only one S1P binding site could be unambiguously fitted in the complex (α1β1 site). In the deoxyHbA-S1P complex, the side-chain of α2Lys99 was in a similar position as 2DN2, consistent with very weak S1P binding as opposed to the ternary deoxyHbA-S1P-2,3-BPG complex. Binding of 2,3-BPG might have increased the affinity of the protein for S1P in the central water cavity. Note that the same concentration of S1P was used during crystallization of both the binary and ternary complexes. Each S1P-associated interaction in the central water cavity was essentially intramolecular in nature (i.e. make interactions with only α1β1 or α2β2) and suggest that central-water cavity bound S1P might not contribute significantly to the stabilization of the T-state structure^29^. This observation indicates that although the affinity of S1P binding to central water cavity of the protein is increased by 2,3-BPG, it is unlikely to cause significant changes to the deoxyHbA conformation.

Interestingly, besides the central water cavity bound S1P as described above, we also found two additional S1P molecules bound in a symmetry-related fashion at the surface of the deoxyHbA-S1P-2,3-BPG complex but not in the deoxyHbA-S1P complex, indicating that 2,3-BPG binding most likely is required for S1P binding at the surface of the protein. Specifically, the phosphate moiety binds close to the α1-heme and located in a highly positive environment formed by α1Arg92, β2Arg40, α1His45, and α1Lys90, as well as with β2Glu43; either making direct salt-bridge/hydrogen-bond interactions and/or water-mediated hydrogen-bond interactions with these residues (Fig. 5f). The S1P amide nitrogen makes water-mediated interaction with α1Lys90 and the α1-heme propionate. The side-chains of both β2Glu43 and α1Lys90 have moved from their native positions to make interactions with the S1P. The highly flexible aliphatic chain snaked along a shallow cavity wall making hydrophobic interactions with the so-called “switch region” residues of β2Phe41, α1Thr41, α1Pro44, β2Leu96, β2His97, as well as with β2-heme, like a molecular sticky tape. The last 3–4 carbon atoms of the aliphatic chain stick out into the bulk solvent (Fig. 5f,g). Similar symmetry related interactions are observed from the β2-heme site to the β1-heme site. As previously noted, the switch region is characterized by significant structural changes during the T to R transition^29,30^, and effectors that prevent these changes are known to decrease Hb affinity for O_2_^29^. These findings raise an intriguing possibility that the surface bound S1P which makes several inter-subunit interactions that involve residues from the switch region serve to stabilize the T-state, and presumably further decrease the T-state affinity for O_2_.

To test this hypothesis, we compared the T-state structures deoxyHbA-S1P, deoxyHbA-S1P-2,3-BPG, native deoxyHbA (PDB code: 2DN2) and native R-state COHbA structure (PDB code: 2DN1)^28^ by superposing their α1α1 dimers (~0.3 Å) and then obtaining the screw rotation angles that are required to superpose the non-superposed α2β2 Hb dimers as a quaternary measure^29,30^. Notably, we found that deoxyHbA-S1P-BPG was further removed from the R-state (15.7°) more than deoxyHbA-S1P (14.8°) and T-state HbA (PDB code: 2DN2) (14.2°). Consistently, the dimer interface β2 F helix/β2FG corner at the switch region show some significant positional differences, with that of deoxyHbA-S1P-BPG further removed from the R-state (Fig. 5h). These observations support our conclusion that 2,3-BPG is required for S1P binding to the protein, especially to the surface of the protein which leads to the protein becoming more tense, and presumably lower affinity for O_2_ compared to either the deoxyHbA or the binary deoxyHbA-S1P complex structures.

## Discussion

Balance of glucose flux between glycolysis and PPP is extremely important in mature erythrocytes and therefore finely tuned. A myriad of studies reported metabolic reprogramming in normal erythrocytes in response to hypoxia through the binding of deoxyHb to cdB3 and subsequently increased cytosolic glycolytic enzymes availability^18–20^. However, in SCD erythrocytes, glucose metabolism is constantly programmed with glycolysis “on” and PPP “off” even at steady normoxia condition^5^, leading to the over production of 2,3-BPG and the shortage of antioxidant glutathione; yet the regulating factors and the underlying mechanisms remain underdetermined before our study. Our findings immediately suggest that glucose in sickle erythrocytes was predominantly metabolized via glycolysis rather than the PPP, as confirmed by tracing experiments. Moreover, genetic deletion of Sphk1 in SCD reprograms glucose metabolism by channeling glucose to PPP instead of glycolysis, which in turn leads to increased NADPH and decreased 2,3-BPG production.

S1P is a versatile bio-active signaling lipid highly abundant in the erythrocytes^25^. Although previous studies showed that S1P levels are increased and contribute to sickling and disease progression in SCD^7^, nothing is known about the underlying mechanism. It is indeed interesting that intracellular S1P at µM concentrations can mediate metabolic reprogramming in SCD by regulating binding of deoxyHb to cdB3. Due to the molar ratio of about 1:300 between cdB3 and Hb^5^, cdB3, not deoxyHb (present at mM concentrations), is the rate-limiting factor in deoxyHb-cdB3 interaction. Interestingly, S1P has an approximately 1:1 molar ratio with cdB3 in normal erythrocytes and an even higher ratio in SCD erythrocytes. Thus, although deoxyHb is expected to be present at a much higher concentration than S1P, it is the concentration of S1P that controls the amount of deoxyHb that binds to cdB3. Besides regulating the sequestration of glycolytic enzymes, it is reasonable to speculate that increased S1P in SCD erythrocytes may also play a role in other cdB3-mediated effects including the binding of S-nitrosohemoglobin and spectrin to cdB3. The former is involved in the nitric oxide (NO) metabolism^31,32^ in erythrocytes while the latter plays a key role in erythrocyte deformability^33^, both of which are important in the pathophysiology of SCD^23,34,35^.

Our data indicate that S1P binds to the surface of 2,3-BPG-Hb and leads to considerable additional conformational change of deoxyHb (by making hydrophobic interactions at the switch interface) to a more T-state character that in part should explain the decreased Hb-O_2_ affinity. It is also notable that the surface-bound S1P could sterically impede diffusion of diatomic ligands (O_2_) into the heme, and in part also decreased Hb-O_2_ affinity. Similar studies have been reported for allosteric effectors that bind and block the heme access to the bulk solvent^29,36^. Although S1P was also observed bound in the central water cavity, the water cavity is known to be a “sink” for several compounds especially those with anionic groups and not all of these compounds show an allosteric effect^29^. Since in the absence of 2,3-BPG, we observed weak binding of S1P at the water cavity and no apparent effect on the protein’s allosteric activity, it is possible that the central water cavity S1P binding is non-specific. Another interesting structural observation is that the last 3–4 carbon atoms of the surface bound S1P do not make any interaction with the protein residue but hang out in the bulk solvent, which could possibly mediate hydrophobic interactions with other proteins, akin to the hydrophobic βVal6 pathogenic mutation involvement in HbS polymerization^1^. S1P, like other effectors of Hb, binds to multiple residues. Mutation of one or multiple residues may result in destabilization of the Hb tetramer. Such study is thus rarely used to ascertain the binding of an effector, but instead structural and/or O_2_ equilibrium studies (Hb-O_2_ binding studies) have been the norm. Importantly, our  structural study showing  surface S1P binding only occurred in the presence of 2,3-BPG binding in the central water cavity is highly suggestive that the surface binding is specific. There are similar reported studies where binding of allosteric effectors to Hb lead to subtle but significant tertiary and/or quaternary structural changes at the heme environment, α1α2 interface, α-cleft or β-cleft^33,37,38^. Such changes have been used to explain the differences in the allosteric activities of these effectors. Notably, effectors that lead to increase in Hb affinity of O_2_ show more relaxed Hb structural features, while the opposite is true for effectors that bind to Hb and decrease its O_2_ affinity for oxygen^29^.

In conclusion, we found that: S1P works collaboratively with 2,3-BPG to cause further conformational changes and stabilize the 2,3-BPG-bound deoxyHbS and HbA to a more enhanced T-state; deoxyHbA or deoxyHbS binds to the membrane protein cdB3, promote release of GAPDH to cytosol and thus channel glucose to glycolysis relative to PPP (Fig. 5i). Altogether, our findings add significant new insight to erythrocyte pathology and physiology and pave the way for novel therapeutic interventions in SCD.

## Methods

### Mice

Berkeley SCD transgenic mice expressing exclusively human HbS were purchased from The Jackson Laboratory (Bar Harbor, ME). *Sphk1* ^−/−^ mice were initially acquired from Dr. Richard L. Proia at the National Institute of Diabetes and Digestive and Kidney Diseases, NIH (Bethesda, MD) and bred in The University of Texas Health Science Center at Houston. Eight-ten weeks old wild-type C57BL6/J mice were purchased from The Jackson Laboratory. All protocols involving animal studies were reviewed and approved by the Institutional Animal Welfare Committee of The University of Texas Health Science Center at Houston. All experiments were performed in accordance with relevant guidelines and regulations from NIH and The University of Texas Health Science Center at Houston.

### Human subjects

Individuals with SCD were identified by hematologists on the faculty of The University of Texas McGovern Medical School at Houston. Subjects participating in this study had no blood transfusion for at least 6 months before blood samples were collected. Normal human subjects were of African descent and were free of hematological disease. The research protocol, which included informed consent from the subjects, was approved by The University of Texas Health Science Center at Houston Committee for the Protection of Human Subjects. Informed consent was obtained from all participants and/or their legal guardians. All experiments involved in human samples were performed in accordance with relevant guidelines and regulations.

### Blood collection and preparation

Mouse blood was collected with heparin or EDTA as an anti-coagulant and centrifuged at 2,000 g for 5 min, followed by aspiration of plasma and white interface. WT erythrocytes were washed once with 5X volume of PBS before storing in the −80 °C freezer. Mature erythrocytes from SCD and *SCD/Sphk1* ^−/−^ mice were isolated using Percoll density centrifugation media (GE Healthcare Life Sciences).

### Isolation of total erythrocytes and treatment of mouse erythrocytes *in vitro*

Blood collected with heparin as an anti-coagulant was centrifuged at 2,000 g for 5 min at room temperature, followed by aspiration of plasma and white interface. Packed mature RBCs were washed 3 times with culture media (F-10 nutrients mix, Life Technologies Thermo Fisher Scientific, Waltham, MA) and re-suspended to 4% hematocrit (HCT). One ml of RBCs were added to each well of a 12-well plate and treated with different concentrations of S1P (Sigma-Aldrich, St. Louis, MO) for 6 hours.

### Sphk1 activity assay

Erythrocyte Sphk1 activity was measured using previously described methods^8^. Briefly, RBCs were lysed in a pH7.4 Tris-HCl buffer containing 1 mM EDTA, 1 mM β-Mercaptoethanol, 0.3% Triton X-100, 50% glycerol and protease and phosphatase inhibitors. Then, the lysates were assayed using 250 µM D-erythro-sphingosine in bovine serum albumin (0.4%) and [γ-^32^P]ATP (10 μCi, 20 mM) containing 200 mM MgCl_2_. Lipids were extracted and then resolved by TLC on silica gel G60 with 1-butanol/methanol/acetic acid/water (80:20:10:20, v/v). The plates were then exposed to phosphor-imaging screening (Bio-Rad) and scanned for radioactive signals as indications of the amount of S1^32^P synthesized.

### Hemoglobin analysis by HPLC

Analysis of different hemoglobin variants was performed by HPLC using Agilent 1100 series HPLC system (Agilent Technologies, CA) and PolyCAT ATM weak cation-exchange column (100 × 4.6-mm, 3 μM, 1500 A; catalog #104CT0315, PolyLC inc., Columbia, MD) as previously described^39^. The chromatographic separation was achieved at 24 °C by a gradient elution of the following mobile phases: mobile phase A contained 40 mM Bis-Tris, 2 mM KCN, pH 6.5; mobile phase B contained 40 mM Bis-Tris, 2 mM KCN, 0.2 M NaCl, pH 6.8. Using a flow rate of 1 ml/min, HPLC column was pre-incubated for 5 min with 18% mobile phase B before sample application. Elution of sample was performed by increasing the mobile phase B to 45% at 8 min, and to 100% at 12 min, then decreasing it back to 18% at 13 min. The column was ready for next sample after re-equilibrating with 18% mobile phase B for 5 min.

### MetHb and COHb measurement

MetHb and COHb were measured using previously described spectrophotometric methods^40,41^. Briefly, oxyHb, COHb, and MetHb standards were prepared from stock standard hemoglobin. Following dilution to 1% (v/v) in distilled water, a 100% saturated solution of oxyhemoglobin was obtained by bubbling oxygen for 10 min, excess O_2_ being removed by bubbling nitrogen for 5 min. Absorbance at wavelength 645 nm was used to calculate concentration of MetHb. For COHb, the absorbance readings were taken at 420 and 432 nm wavelengths. The absorbance ratio (Ar) was calculated as: Ar = A420/A432. Factors F1, F2, and F3, were calculated by subjecting the 100% COHb and 100% O_2_Hb samples to the same procedure. Thus, F1 = A(O_2_Hb)432/A(O_2_Hb)420, F2 = A(COHb)432/A(O_2_Hb)420, and F3 = A(COHb)432/A(O_2_Hb)420. The % COHb was calculated using the following equation:

$$ \% {\rm{COHb}}=[1-({\rm{Ar}}\times {\rm{F}}1)]/[{\rm{Ar}}({\rm{F}}2-{\rm{F1}})-{\rm{F}}3+1].$$

### Glucose uptake assay

Blood was collected with heparin as an anticoagulant and centrifuged at 2,400 g for 5 min at room temperature, followed by aspiration of plasma and buffy coat. Packed erythrocytes were purified using percoll gradients to remove reticulocytes. The mature erythrocytes were washed three times with PBS and re-suspended to 4% hematocrit. The uptake assay started with transferring 54 µl of erythrocyte suspension to an Eppendorf tube with 6 µl C_14_-glucose (PerkinElmer, Waltham, MA) master mix (50 mM adenosine with 1 mCi∙ml^−1^ C_14_-glucose in PBS) to get a final glucose concentration of 5 mM. The uptake was performed for up to 120 min and stopped by adding 100 ml cold stop solution (0.9% saline), then centrifuged at 2,400 g for 5 min. The supernatant was withdrawn and the erythrocyte pellet was lysed in 60 µl water and the lysate was spread on a glass microfiber filter (GE Healthcare Life Sciences, catalogue number: 1825-025), heat-dried for counting of C_14_ isotope using a scintillation counter (LKB WALLAC 1209 EACKBETA Liquid Scintillation Counter, LKB Instruments, Victoria, Australia). Also, 54 µl of erythrocyte suspension (washed and re-suspended to 4% hematocrit as mentioned above) was aliquoted for total protein measurement using Pierce BCA Protein Assay kit (Thermo Scientific, catalogue #: 23225, Rockford, IL, USA).

### Metabolomics Profiling

#### Metabolomics extraction

RBCs (100 µl) and plasma samples (20 µl) were immediately extracted in ice-cold lysis/extraction buffer (methanol:acetonitrile:water 5:3:2) at 1:9 and 1:25 dilutions, respectively. Samples were agitated at 4 °C for 30 min, and then centrifuged at 10,000 g for 15 min at 4 °C. Protein pellets were discarded, and supernatants were stored at −80 °C prior to metabolomics analyses^42^.

#### Metabolomics analysis

Ten µl of RBC extracts were injected onto a UHPLC system (Ultimate 3000, Thermo, San Jose, CA, USA) and run on a Kinetex XB-C18 column (150 × 2.1mm, 1.7 µm particle size - Phenomenex, Torrance, CA, USA) using a 3 min isocratic flow (5% acetonitrile, 95% water, 0.1% formic acid) at 250 µl/min or a 9 min linear gradient (5–95% acetonitrile with 0.1% formic acid at 400 µl/min). The UHPLC system was coupled online with a Q Exactive mass spectrometer (Thermo, Bremen, Germany), scanning in Full MS mode (2 µscans) at 70,000 resolution in the 60–900 m/z range, 4 kV spray voltage, 15 sheath gas and auxiliary gas, operated in negative and then positive ion mode (separate runs). Calibration was performed before each analysis using positive and negative ion mode calibration mixes (Pierce, Rockford, IL, USA) to ensure sub ppm error of the intact mass. Metabolite identifications were assigned using the software Maven (Princeton, NJ, USA), upon conversion of raw files into mzXML format through MassMatrix (Cleveland, OH, USA). The software allows for peak picking, feature detection and metabolite assignment against the KEGG pathway database. Assignments were further confirmed against chemical formula determination (as gleaned from isotopic patterns and accurate intact mass), and retention times against a >750 standard compound library (Sigma-Aldrich, St. Louis, MO, USA; IROA Tech, Bolton, MA, USA)^42^.

### Metabolic flux analysis

For the glucose flux experiment, RBCs were cultured in HEPES buffer with 6 mM D-Glucose-1,2,3-^13^C_3_ (Sigma Aldrich)^20,43^. RBCs were extracted and processed as described above. Packed mature RBCs were washed 3 times with HEPES buffer and re-suspended to 4% hematocrit (HCT). One ml of RBCs was added to each well of a 12-well plate and pretreated for 30 min before sample collection started. Flux analysis was performed by determining the integrated peak areas of isotopologues +2.0068 and +3.0102 Da of lactate, glucose, and G3P in negative ion mode through the software Maven (Princeton, NJ, USA).

### S1P quantification and sphingolipids analysis

Validation and quantitative analyses for sphingolipids and S1P were performed using a Thermo Vanquish UHPLC system coupled to a Thermo Q Exactive mass spectrometer and determined against commercial standard compounds sphingosine 1-phosphate (>95% pure - no. S9666, Sigma Aldrich, St. Louis, MO, USA) and sphingosine-1-phosphate-d7 (>99% pure - no. 860659 P – Avanti Lipids Polar Inc, Alabaster, AL, USA) or sphingosine/ceramide deuterated mixes (LM-6002 - Avanti Lipids Polar Inc, Alabaster, AL, USA) within the linearity range, as determined through external calibration curves across 5 orders of magnitude. Samples were diluted 1:10 with methanol:acetonitrile:water (5:3:2) containing 100 nM S1P-d7 or sphingosine/ceramide mixes, then agitated and centrifuged as described above. Supernatant (10 µl per injection) was analyzed using both a 4 and 9 minute gradient of 50–95% acetonitrile containing 0.1% formic acid (400 µl/min) and a Kinetex C18 column (150 × 2.1 mm, 1.7 µm – Phenomenex) held at 35 °C. The mass spectrometer was operated in positive ion mode at 70,000 resolution, scan range of 90–1350 m/z, sheath gas 25, auxiliary gas 5.

Quantification was performed by exporting integrated peak area values for endogenous and heavy S1P, sphingosine or ceramides. Absolute quantitation was determined according to the formula: [light] = Peak Area (Light)/(Heavy)*[Heavy]*dilution factor (10 for red blood cells, 25 for plasma). Results were imported into GraphPad Prism 5.0 (GraphPad Software Inc., La Jolla, CA, USA) for statistical analysis (One way ANOVA with Tukey multiple column comparison test; significance threshold for p-values < 0.05).

### Morphology study of erythrocytes

Blood smears were made using 1% glutaradehyde fixed cultured human or tail blood from bone marrow transplanted mice. Blood smears were stained by WG16-500ml kit (Sigma-Aldrich) for sickle cell. Blood smears stained by these procedures were observed using the 40x objective of an Olympus BX60 microscope. Areas where red blood cells do not overlap were randomly picked, at least 10 fields were observed and 1000 red blood cells including sickle cells were counted. The percentages of sickle cells in red blood cells were calculated.

### Hemolytic analysis

The hemoglobin in mouse plasma was quantified by ELISA kits following instructions provided by the vendor (BioAssay Systems, Hayword, CA).

### Mouse organ isolation and histological analysis

Mice were anesthetized and organs were isolated and fixed with 10% paraformaldehyde in PBS overnight at 4 °C. Fixed tissues were rinsed in PBS, dehydrated through graded ethanol washes, and embedded in paraffin. 5μm sections were collected on slides and stained with hematoxylin and eosin (H&E). The semi-quantitative analysis of histological changes was conducted as previously described using a computerized program^7^. Ten digital images were taken from each H&E stained mouse tissue section at 20X magnification from different areas. The congestion, necrosis or cysts on sections were identified according to their structure and color. Briefly, the dark red color was chosen for quantification of congestion and it was performed on 10 fields/mouse tissue sections at 20× magnification using software analysis (Image Pro Plus 4.0; Media Cybernetics, Bethesda, MD, USA). Additionally, the areas of necrosis in the livers and cysts in the renal cortex were first manually marked by a magical pen tool available in Adobe Photoshop Program. Then the quantification was conducted on 10 fields/mouse tissue sections at 20× using software analysis (Image Pro Plus 4.0; Media Cybernetics, Bethesda, MD, USA). The whole areas of each image were considered as 100%. The percentage of pathological areas to whole area of image was recorded.

### Measurement of life span of erythrocytes in SCD Tg mice

Erythrocytes were labeled *in vivo* by using N-hydroxysuccinimide (NHS) biotin and the life span of circulating red blood cells was measured as described^7^. Specifically, 50 mg/kg of NHS biotin was injected into the retro-orbital plexus of SCD mice (prepared in 100 μl sterile saline just prior to injection; initially dissolved at 50 mg/mL in dimethyl sulfoxide). Blood samples (5 μl) were collected the first day after biotin-injection from tail vein by venipuncture to determine the percentage of erythrocytes labeled with biotin. Subsequently, 5 μl of blood were obtained by tail vein venipuncture on day 1, 3, 5 and 7 for measurement of biotinylated erythrocytes. The percentage of biotinylated erythrocytes was calculated by determining the fraction of peripheral blood cells labeled with Ter-119 (to identify erythrocytes) that were also labeled with a streptavidin-conjugated fluorochrome by flow cytometry

### 2,3-BPG analysis and erythrocyte O_2_ release capacity (P50) measurement

RBC 2,3-BPG was isolated as indicated before and quantified by a commercially available kit (Roche, Nutley, NJ)^6^. For P50 measurement in intact cells, 10 μl of whole blood aliquot were mixed with 4.5 ml Hemox Buffer (TCS Scientific Corporation, PA), 10 μl anti-foaming reagent ((TCS Scientific Corporation, PA) and 20 μl 22% BSA in PBS; for P50 measurement of Hb with or without 2,3-BPG or S1P, the system was prepared first as indicated above. Then, the mixture was then injected in the Hemox Analyzer (TCS Scientific Corporation, PA) for measurement of O_2_ equilibrium curve at the temperature of 37 °C.

### Measurement of NADPH

RBC NADPH was quantified by a commercially available kit (Sigma-Aldrich). Briefly, 10 µl RBCs were used for each assay. NADPH was extracted with 800 µl of NADP/NADPH Extraction Buffer and placed on ice for 10 minutes, then centrifuged the samples at 10,000 g for 10 minutes to remove insoluble material. Then, 10KDa molecular weight cut-off columns were used to filter out enzymes in the lysate. The filtered solution was then applied to the measurement of NAPDH through a chain colorimetric reaction and the read-outs were detected by spectrophotometer.

### Isolation of RBC cytoplasm and measurement of GAPDH activity

RBCs were lysed by freeze and thaw in 10 volume of 5 mM cold phosphate buffer (pH 8.0) and vortexed. RBC membrane was removed by centrifuged at 20,000 g for 20 minutes at 4°C. The supernatant was saved and used to measure cytosolic GAPDH activity by KDalert GAPDH assay kit (Life technologies).

### Western Blot detection of GAPDH in erythrocytes

Pelleted erythrocytes were first frozen and then thawed in 20-fold volume of 5 mM phosphate buffer containing 150 mM NaCl, protease inhibitors (Roche) and phosphatase inhibitors (Sigma). Then, 200 µl were isolated and used as total lysate. The rest were centrifuged with 20,000 g for 20 min. Supernatant were removed and pellets were washed for four times before dissolving in the same buffer with 1% Triton X-100. 50 µg of membrane protein and 150 µg total protein were loaded for western blot detection of membrane bound and total GAPDH using monoclonal anti-GAPDH antibody (Sigma-aldrich) (1:1000 in blocking buffer).

### Membrane bound heme measurement

Heme bound on membrane was measured previously described^44^ with minimum modification. In brief, nonporous slica beads with 3.15 µm diameter (Bangs Laboratories, IN) were pretreated as previously described^44^. Human or mouse ghost cells were prepared as follow: heparin-blood was centrifuged at 2,400 g for 5 minutes. The plasma and buffy coat were removed. The pellet was washed twice with Phosphate Buffered Saline (PBS). The cells were lysed in 5 mM phosphate buffer (pH8.0), centrifuged at 18,000 g for 15 minutes. The supernatant was removed and the pellet was washed in phosphate buffer for 7 times to obtain ghost cells. The beads were coated by ghost cells to produce inside-out membrane (IOM). The IOM was washed 6 times with 5 mM phosphate buffer (PB, pH 8.0). Packed 5 × 10^9^ IOM beads were added 100 μl 5 mM PB (pH7.4) with 100 μM hemoglobin, varied concentration S1P. Beads were incubated at 37°C for 10 minutes, centrifuged at room temperature for 1 minute at 500 g. The supernatant was transferred to new tube for GAPDH activity assay. Pellet beads were washed 6 times with PB (pH7.4). Beads were added to 100 µl of concentrated formic acid (Sigma-Aldrich), vortexed for 5 minutes. The beads were centrifuged at 2000rpm for 2 minutes. 80 µl of the supernatant was transferred to a new 1.5 ml tube and added 400 µl 5 M NaOH. The heme concentration was determined at 398 nm wavelength as described and normalized to protein concentration. Human Hb A was used as standards for heme assay.

### S1P beads pull down assay

Two µg of total erythrocyte lysate from normal individuals was adjusted to 100 µL using lysis buffer (20 mM PIPES, 150 mM NaCl, 1 mM EGTA, 1% V/V Triton-X-100, 1.5 mM MgCl_2_ and 1 mM Naorthovanidate, 0.1% SDS, 1X protease inhibitors (Roche Applied) pH7.4). Approximately 100 µl of various lipids conjugated to agarose beads including S1P-agarose beads, lysophosphatic acid-beads or sphingosine-beads (Echelon Biosciences Inc, Salt Lake City, UT) were washed twice with lysis buffer. The lysates were incubated with beads overnight at 4°C with constant gentle rotation. Protein-bound beads were washed by wash buffer (10 mM HEPES pH 7.4, 150 mM NaCl, 0.25% NP-40) for 6 times. Washed beads were added 50 µL of 2× Laemmli buffer (Sigma-Aldrich) and heated at 100°C for 5 minutes. Beads were centrifuged at 5000 g for 5 minutes and supernatants (eluted proteins) were separated by SDS-PAGE, transblotted to nitrocellulose membrane. Sickle Hemoglobin on the membrane was probed with anti-human hemoglobin antibody (Santa Cruz, CA). Immunoreactive bands were visualized by ECL using secondary antibodies conjugated with horseradish peroxidase and Super-signal West Pico chemiluminesence substrate (Piere).

### Crystal Structural studies

Freshly prepared solution of S1P in methanol was incubated with deoxygenated Hb (40 mg/mL deoxyHb) with and without freshly prepared solution of 2,3-BPG in water for 60 minutes at Hb tetramer: 2,3-BPG:S1P molar ratio of 1:5:5 or Hb tetramer:S1P molar ratio of 1:5. The binary (deoxyHb-S1P) and ternary (deoxyHb-S1P-2,3-BPG) complex solutions were then crystallized with 0.2 M sodium acetate trihydrate, 0.1 M sodium cacodylate trihydrate, pH 6.5 and 30% PEG 8000 using the batch method as previously described^45^. Crystals were cryo-protected with mother liquor and glycerol (3:1 ratio) prior to diffraction data collection at 100 K with a Rigaku IV ++ image plate detector using a CuKα X-rays (λ = 1.54 Å) from a MicroMax-007 source fitted with Varimax Confocal optics (Rigaku, The Woodlands, TX). The two complexes crystalized in orthorhombic space group P2_1_2_1_2, each with one tetramer per asymmetric unit. The datasets were processed with the d*trek software (Rigaku) and the CCP4 suite of programs^46^.

The deoxyHb-S1P structure was first determined using molecular replacement method with Phenix v.1.8^47^, with the native deoxyHb structure, deoxyHb (2DN2)^28^ and refined with both Phenix^47^ and the CNS programs^38^. Model building and correction were carried out using the graphic program COOT^48^. The refined-S1P structure was then used as a starting model to refine the deoxyHb-S1P-BPG complex structure. The deoxyHb-S1P refines to Rfactor/Rfree of 22.4/27.9% at 2.4 Å, while deoxyHb-S1P-2,3-BPG refines to 18.2/21.1% at 1.8 Å. A significant number of the low-resolution reflections in the ternary deoxyHb-S1P-BPG complex were characterized by high mosaicity, which could have contributed to the large difference in the Rfactor and Rfree. The atomic coordinate and structure factor files have been deposited in the RCSB Protein Data Bank with accession codes 5KSJ for deoxyHb-S1P and 5KSI for deoxyHb-S1P-2,3-BPG. Detailed crystallographic and structural analysis parameters are reported in Table 1.

**Table 1:** Crystallographic data for deoxyHbA-S1P-2,3-BPG and deoxyHbA-S1P complex structures.

	deoxyHbA-S1P-2,3-BPG	deoxyHbA-S1P
**Data Collection Statistics**		
Space group	P2_1_2_1_2	P2_1_2_1_2
Cell dimensions (Å)	95.94, 98.08, 65.14	97.56, 95.15, 64.98
Molecules/asymmetric unit	1 tetramer	1 tetramer
Resolution (Å)	29.42–1.80 (1.86–1.80)	29.33–2.40 (2.49–2.40)
No. of measurements	221938 (21321)	119348 (10341)
Unique reflections	54124 (5483)	24236 (2293)
I/sigma I	11.5 (3.4)	9.4 (3.1)
Completeness (%)	93.9 (96.4)	96.6 (96.8)
Rmerge (%)^a^	7.6 (38.0)	12.0 (39.8)
**Refinement Statistics**		
Resolution limit (Å)	29.42–1.80 (1.88–1.80)	29.08–2.40 (2.51–2.40)
Sigma cutoff (F)	0.0	0.0
No. of reflections	54123 (6869)	24093 (2969)
Rfactor (%)	18.1 (29.6)	22.4 (34.3)
Rfree (%)^b^	21.8 (32.7)	27.9 (38.3)
**Rmsd standard geometry**		
Bond-lengths (Å)/−angles (°)	0.010/1.5	0.000/1.6
Dihedral angles		
Most favored/allowed regions	96.8/3.2	92.4/6.7
Average B-Factors		
All atoms/Protein/Heme	23.5/19.3/16.9	43.2/42.6/41.6
Water/S1P/2,3-BPG	40.4/73.5/58.9	47.5/96.2

Values in parentheses refer to the outermost resolution bin. ^a^ *R*_merge_ = Σ_*hkl*_Σ_i_|*I*_i_(*hkl*) − <*I*(*hkl*)>|/Σ_*hkl*_Σ_i_ *I*_i_(*hkl*). ^b^R_free_ was calculated from 5% randomly selected reflection for cross-validation. All other measured reflections were used during refinement.

### Statistical analysis

All data were presented as mean ± standard deviation and analyzed statistically using GraphPad Prism 5 software (GraphPad Software). The significance of differences among two groups was assessed using Two-tailed Student’s *t*-test. Differences between the means of multiple groups were compared by one-way analysis of variance (ANOVA) or two way ANOVA, followed by a Turkey’s multiple comparisons test. A *P* value of less than 0.05 was considered significant.

## Electronic supplementary material

SupplementaryInformation
