# Supplementary material for: Structural and Functional Insight of Sphingosine 1-Phosphate-Mediated Pathogenic Metabolic Reprogramming in Sickle Cell Disease
Source: Sci Rep. 2017 Nov 10;7:15281. doi: 10.1038/s41598-017-13667-8 (PMC5681684; doi:10.1038/s41598-017-13667-8)

Supplementary Information

Structural and Functional Insight of Sphingosine 1-Phosphate-Mediated Pathogenic Metabolic Reprogramming in Sickle Cell Disease

By

Kaiqi Sun[1,2], Angelo D'Alessandro[3], Mostafa H Ahmed[4], Yujin Zhang[1], Anren Song[1], Tzu-Ping Ko[4], Travis Nemkov[3], Julie A. Reisz[3], Hongyu Wu[1], Morayo Adebiyi[1,2], Zhangzhe Peng[1,5], Jing Gong[1], Hong Liu[1,2], Aji Huang[1], Yuan Edward Wen[1], Alexander Q. Wen[1], Vladimir Berka[1,7], Mikhail V. Bogdanov[1], Osheiza Abdulmalik[6], Leng Han[1], Ah-lim Tsai[7], Modupe Idowu[7], Harinder S. Juneja [7], Rodney E. Kellems[1,2], William Dowhan[1], Kirk C. Hansen[3],
Martin K. Safo[4], and Yang Xia*[1,2,5]

[1]Department of Biochemistry and Molecular Biology, [2]Graduate School of Biomedical Science, [7]Department of Internal Medicine-Hematology, The University of Texas Health Science Center at Houston, Houston, TX 77030, USA; [3]Department of Biochemistry and Molecular Genetics, University of Colorado School of Medicine, Aurora, CO 80045, USA; [4]Department of Medicinal Chemistry, and The Institute for Structural Biology, Drug Discovery and Development, School of Pharmacy, Virginia Commonwealth University, Richmond, VA 23298, USA; [5]Department of Nephrology, Xiangya Hospital, Central South University, Changsha, Hunan 410008, China;[6]Division of Hematology, The Children's Hospital of Philadelphia, Philadelphia, PA 19104, USA;

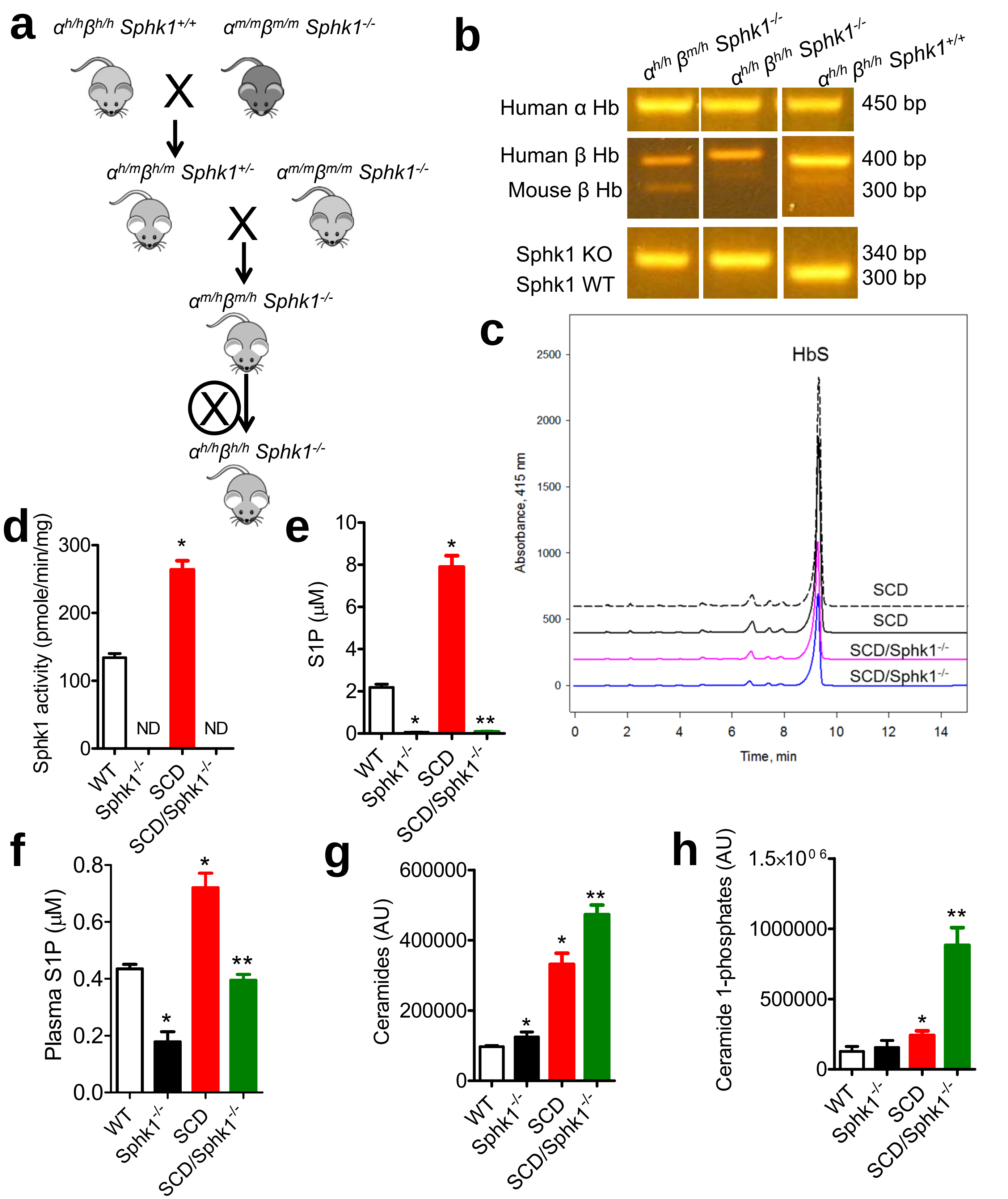

Supplementary Figure 1: Generation and confirmation of *SCD/Sphk1*$^{-/-}$ mice. (**a**) Schematic plot demonstrating the mating strategy employed to generate *SCD/Sphk1*$^{-/-}$ mice. (**b**) PCR analysis of genomic DNA detecting α,β Hb genes and *Sphk1* gene. (**c**) HPLC analysis of Hb composition in SCD and *SCD/Sphk1*$^{-/-}$ mice. Erythrocyte Sphk1 activity (**d**), erythrocyte (**e**) ,plasma (**f**) S1P levels, and erythrocyte ceramides (**g**) and ceramide 1-phosphates (**h**) in WT, *Sphk1*$^{-/-}$,SCD and *SCD/Sphk1*$^{-/-}$ mice. Mean ± s.e.m; N=5 for each group; *p<0.05 versus WT, **p<0.05 versus SCD, Student's *t*-test.

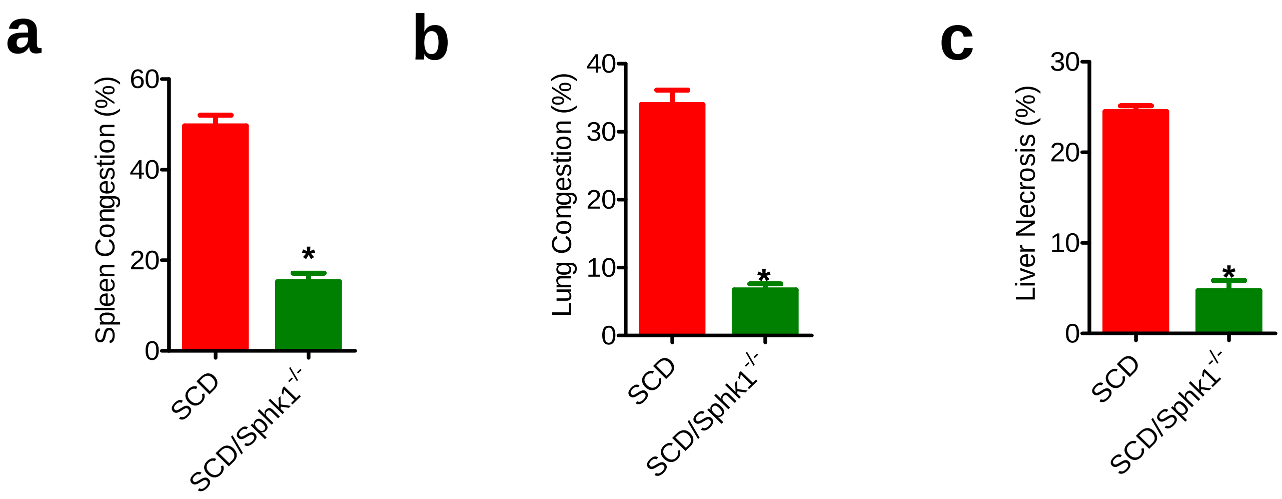

Supplementary Figure 2: Semi-quantitative analysis of H&E stained sections showed attenuated organs injury in *SCD/Sphk1*$^{-/-}$ mice. Decreased spleen congestion (**a**), lung congestion (**b**) and liver necrosis in (**c**) in *SCD/Sphk1*$^{-/-}$ mice compared to SCD mice. Mean ± s.e.m; n=5 for each group; *p<0.05 versus SCD, Student's *t*-test.

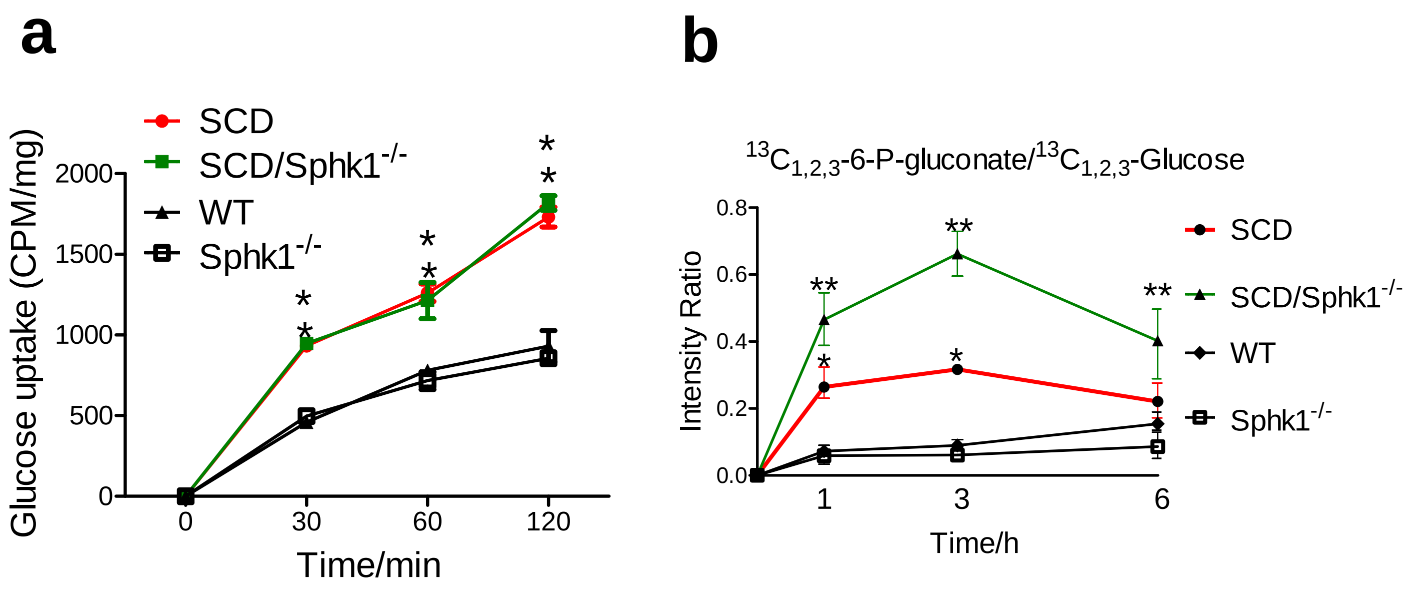

Supplementary Figure 3: Increased PPP in *SCD/Sphk1*$^{-/-}$ erythrocytes is independent of glucose uptake. Glucose uptake(**a**) and ratios of $^{13}C_{1,2,3}$-6-P-gluconate/$^{13}C_{1,2,3}$-Glucose in WT, *Sphk1*$^{-/}$, SCD and *SCD/Sphk1*$^{-/-}$ erythrocytes.Mean ± s.e.m; n=3~5 for each group; *p<0.05 versus WT; **p<0.05 versus SCD, Student's *t*-test.

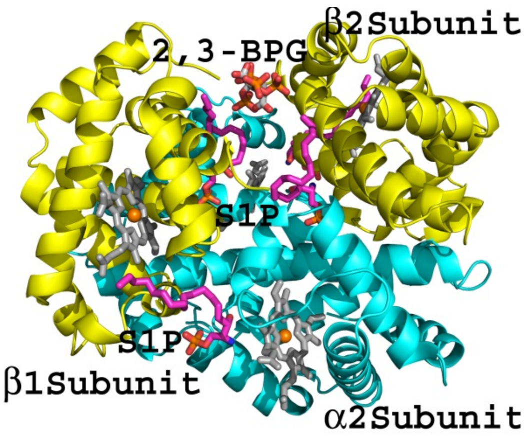

Supplementary Figure 4: Ribbon diagram of tetrameric structure of Hb with bound 2,3-BPG at the β-cleft, 2 molecules of S1P bound in the central water cavity, and two other molecules of S1P bound at the surface of the protein. The α-subunits are colored in cyan and β-subunits in yellow. The small molecules are shown in sticks. This figure is similar to Fig.5e but in different orientation.

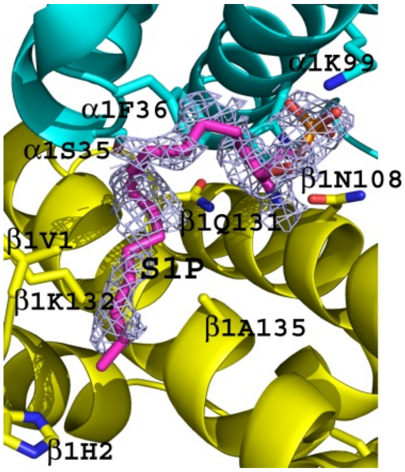

Supplementary Figure 5: Binary structure of the central water-cavity bound S1P.

Supplementary Table 1: Complete Blood Count of WT, SCD and *SCD/Sphk1*$^{-/-}$ mice. Values shown represent the mean ± SEM (n = 5); *p<0.05 versus SCD, Student's *t*-test.

	WT	SCD	*SCD/Sphk1*$^{-/-}$
**Erythrocyte**			
RBC (M/µl)	9.42 ± 0.78	5.26 ± 0.39	6.79±0.59*
Hb (g/dl)	14.5 ± 0.36	7.15 ± 0.26	9.83 ± 0.55*
HCT (%)	44.8 ± 1.05	28.53 ± 0.98	34.55 ± 0.47*
MCV (fl)	49.8 ± 2.33	44.53 ± 0.97	50.60 ± 3.47*
MCH (pg)	15.23 ± 0.17	10.83 ± 0.31	14.06 ± 0.70*
MCHC (g/dl)	29.9 ± 1.22	25.33 ± 1.21	26.68 ± 0.88
RDW (%)	18.5 ± 4.35	32.47 ± 5.47	23.58 ± 3.54*
**Leukocyte**			
WBC (k/µl)	4.90 ± 2.98	18.56 ± 5.10	5.12 ± 2.16*
NE (k/µl)	1.44 ± 1.74	11.25 ± 2.37	2.57 ± 1.49*
LY (k/µl)	4.55 ± 3.13	6.81 ± 2.04	2.15 ± 1.50*
MO (k/µl)	0.41 ± 0.10	0.45 ± 0.40	0.35 ± 0.24
EO (k/µl)	0.14 ± 0.05	0.04 ± 0.006	0.05 ± 0.03
BA (k/µl)	0.03 ± 0.005	0.013 ± 0.005	0.006 ± 0.008

Values shown represent the mean ± SEM (n = 5); *p<0.05 versus SCD, Student's *t*-test.

WT and Sphk1$^{-/-}$ total lysate GAPDH

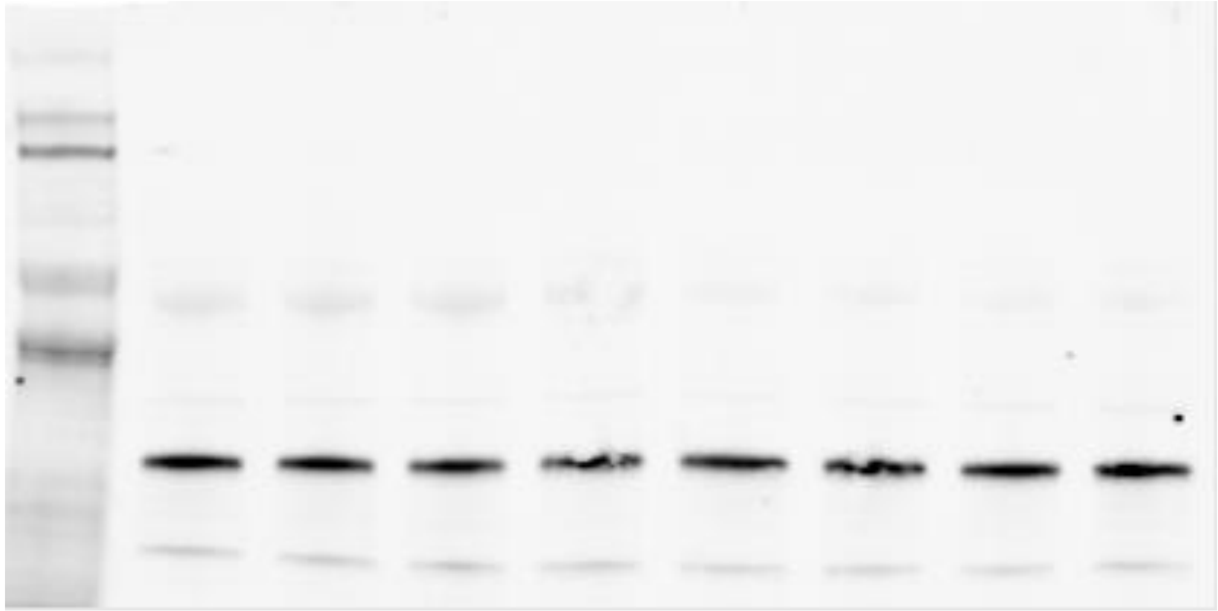

WT and Sphk1$^{-/-}$ total lysate β-actin

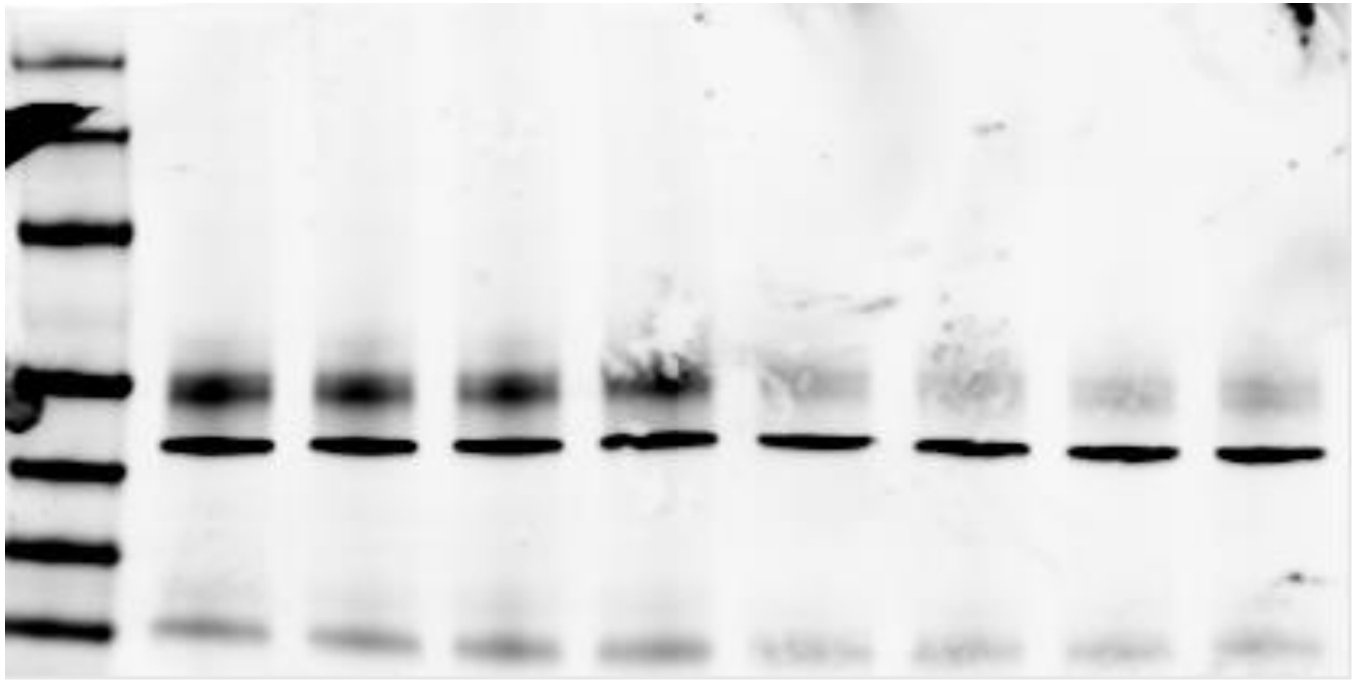

WT and Sphk1$^{-/-}$ membrane GAPDH

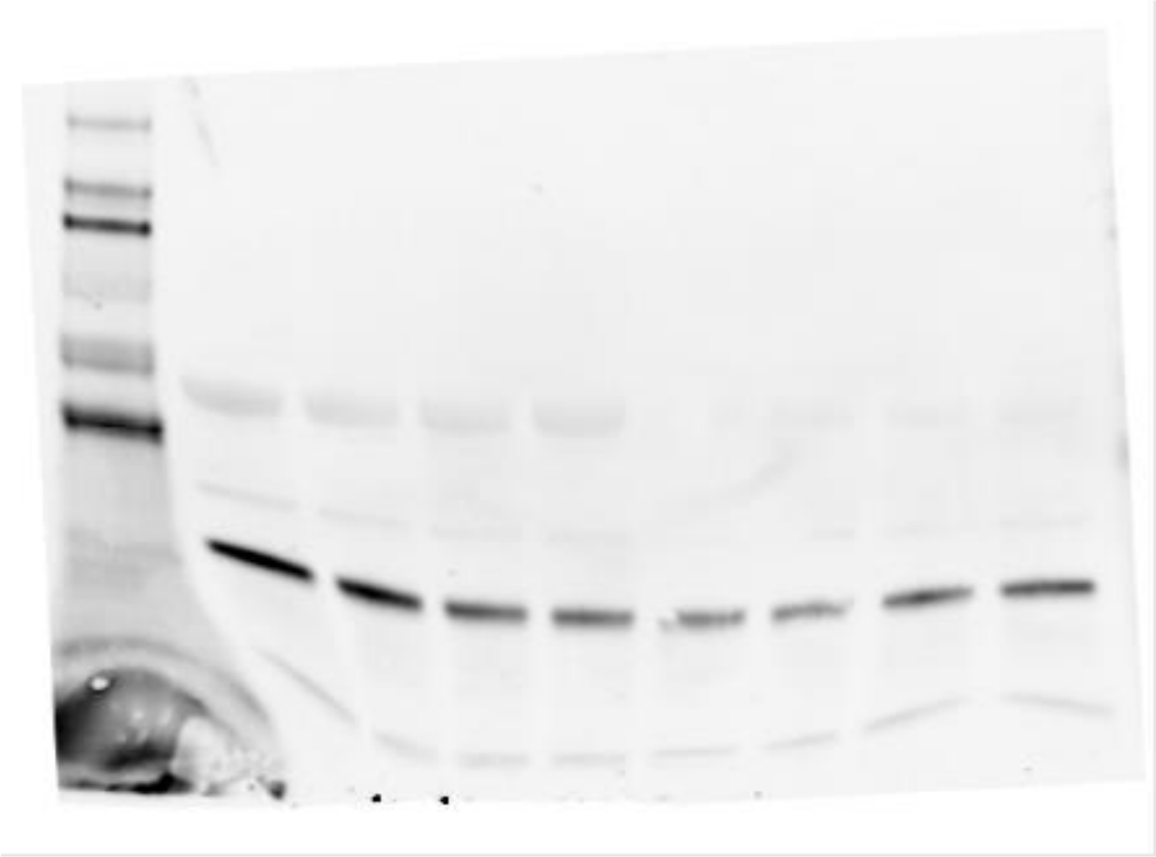

WT and Sphk1$^{-/-}$ membrane β-actin

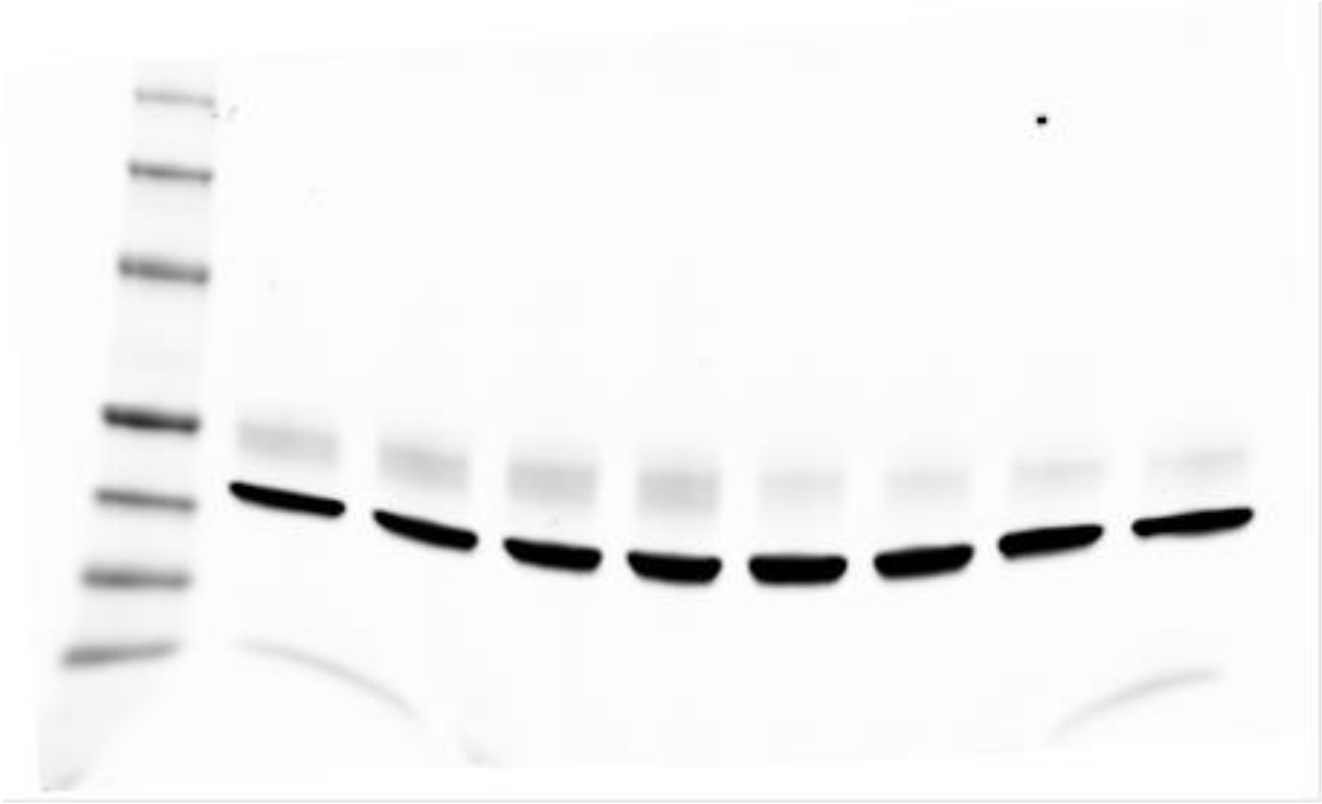

SCD/Sphk1$^{-/-}$ and SCD total lysate GAPDH

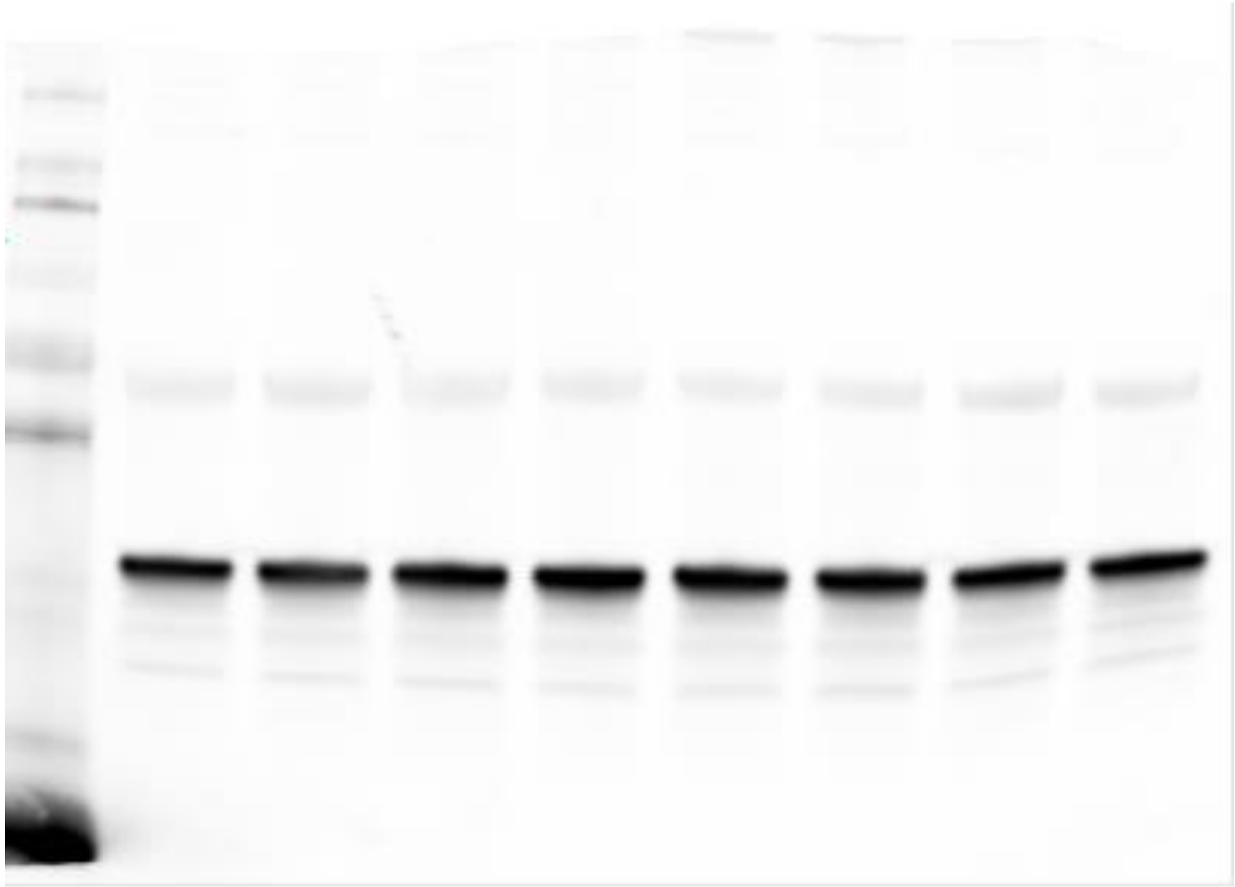

SCD/Sphk1$^{-/-}$ and SCD total lysate β-actin

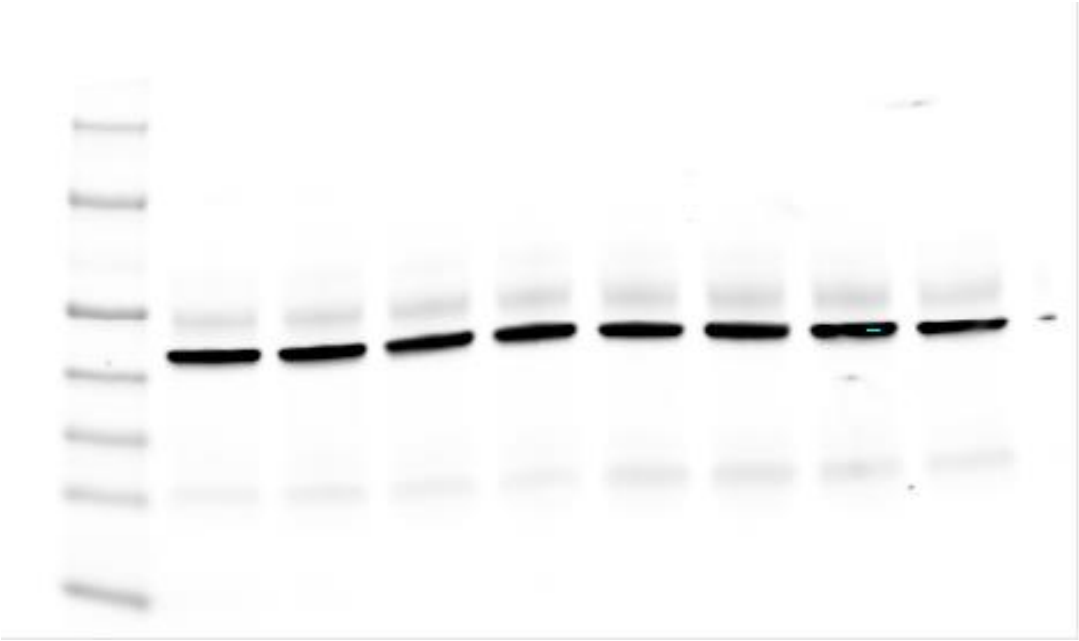

SCD/Sphk1$^{-/-}$ and SCD membrane GAPDH

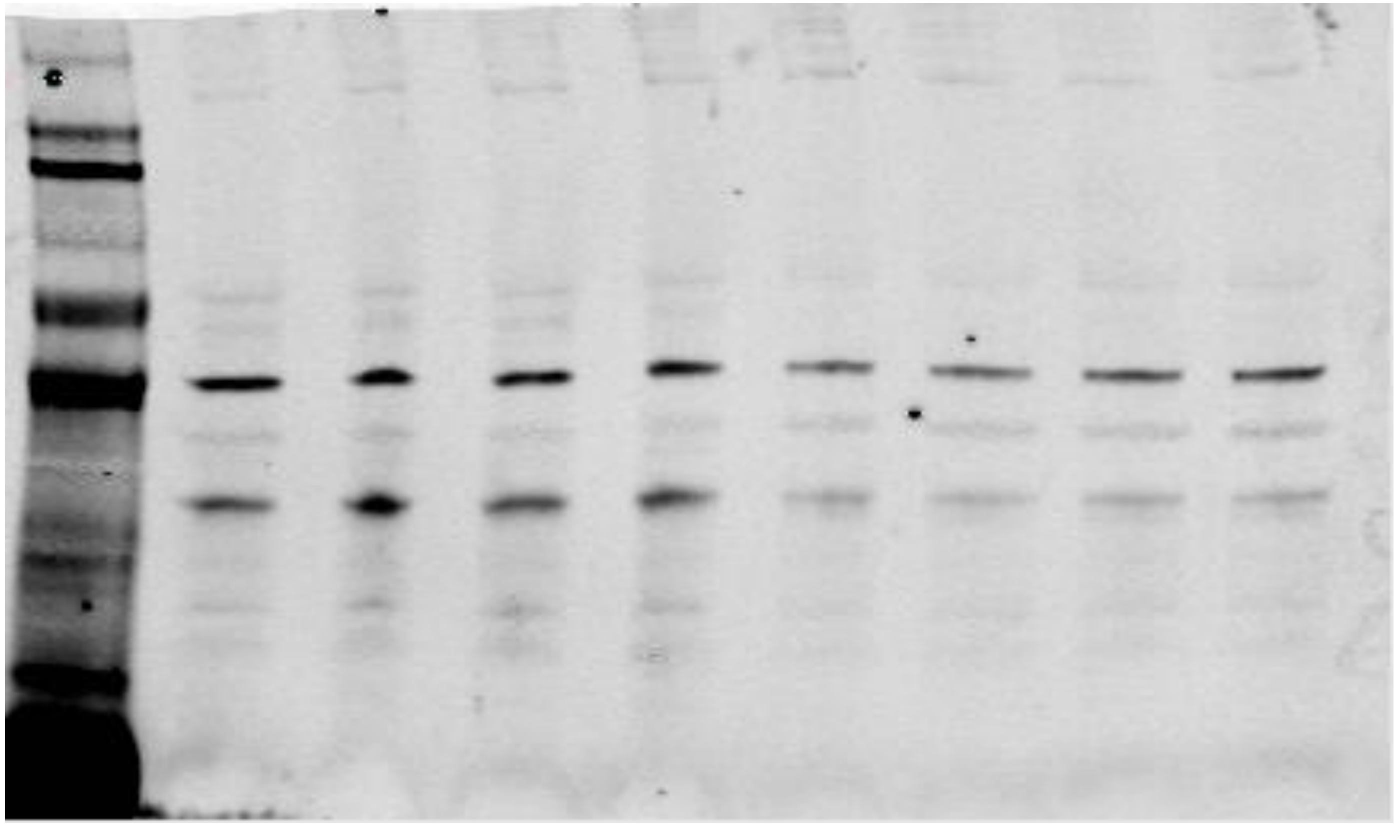

SCD/Sphk1$^{-/-}$ and SCD membrane β-actin

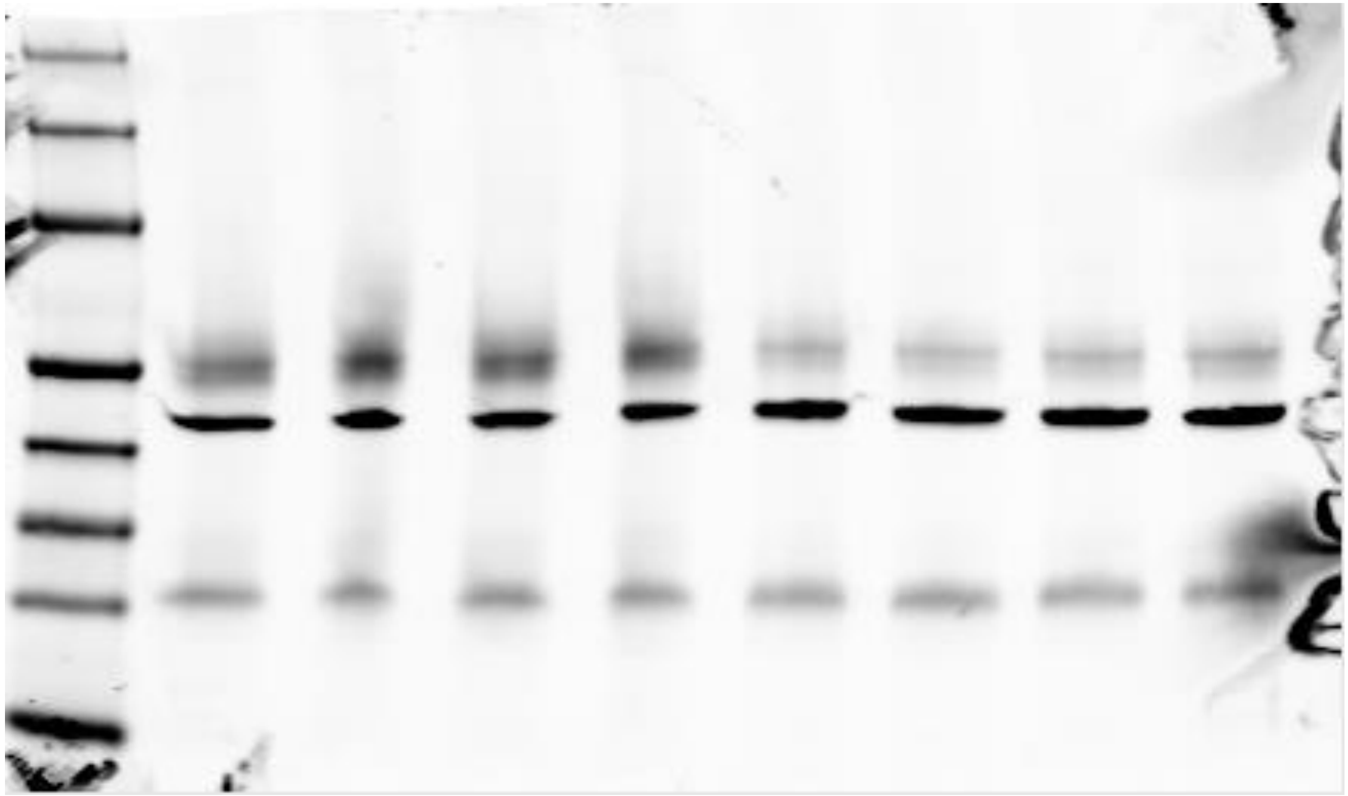

S1P-beads pull-down

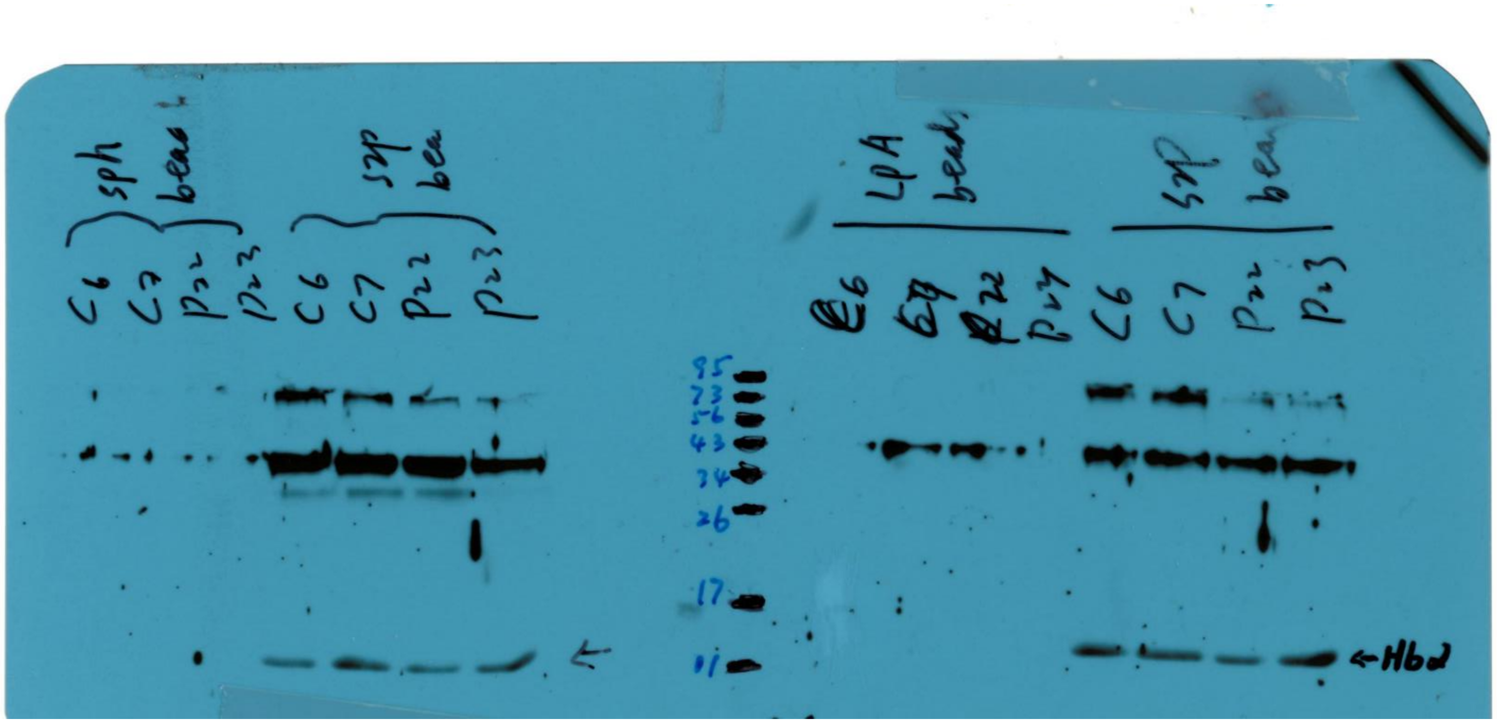

Supplement: Supplementary file 1 — SupplementaryInformation [file 41598_2017_13667_MOESM1_ESM.pdf]
